# Supplementary material for: Antenatal and postpartum prevention of Rh alloimmunization: A systematic review and GRADE analysis
Source: PLoS One. 2020 Sep 10;15(9):e0238844. doi: 10.1371/journal.pone.0238844 (PMC7482964; doi:10.1371/journal.pone.0238844)
Supplement: S1 File — (DOCX) [file pone.0238844.s001.docx]

# S1. Search strategies (RCT and observational)

## RCT search

**Medline and CENTRAL, DARE and Cochrane’s Database of Systematic Reviews**

1. exp pregnancy/

2. exp pregnancy complications/

3. exp pregnancy trimesters/

4. pregnan*.tw.

5. prenatal care/

6. postnatal care/

7. ((prenatal or pre-natal or postnatal or post-natal or antenatal or ante-natal or postpartum or post-partum) adj3 (care* or servic*)).tw.

8. (abortion* or miscarr* or trauma* or terminat* or haemorrhag* or hemorrhag*).tw.

9. ((fetal or neonatal or neo-natal) adj3 death*).tw.

10. or/1-9

11. Rh Isoimmunization/

12. "Rho(D) Immune Globulin"/ or Rh-Hr Blood-Group System/

13. ((rh or rhesus) adj4 (senti?ation* or isoimmuni?ation*)).tw.

14. (prophylax* adj5 (Rh or anti-D)).tw.

15. or/11-14

16. randomized controlled trial.pt.

17. controlled clinical trial.pt.

18. randomized.ti,ab.

19. placebo.ti,ab.

20. drug therapy.sh.

21. randomly.ti,ab.

22. groups.ti,ab.

23. or/16-22

24. 10 and 15 and 23

**Embase**

1. exp pregnancy/

2. exp pregnancy complication/

3. pregnan*.tw.

4. exp prenatal care/

5. postnatal care/

6. exp postnatal care/

7. ((prenatal or pre-natal or postnatal or post-natal or antenatal or ante-natal or postpartum or post-partum) adj3 (care* or servic*)).tw.

8. (abortion* or miscarr* or trauma* or terminat* or haemorrhag* or hemorrhag*).tw.

9. ((fetal or neonatal or neo-natal) adj3 death*).tw.

10. or/1-9

11. exp rhesus isoimmunization/

12. exp rhesus D antibody/ or exp blood group rhesus system/

13. ((rh or rhesus) adj4 (senti?ation* or sensibili?ation* or iso-immuni?ation* or isoimmuni?ation*)).tw.

14. (prophylax* adj5 (Rh or anti-D)).tw.

15. or/11-14

16. clinical trial/

17. controlled clinical trial/

18. randomized controlled trial/

19. multicenter study/

20. exp randomization/

21. crossover procedure/

22. single blind procedure/

23. double blind procedure/

24. placebo/

25. randomi?ed controlled trial$.tw.

26. rct.tw.

27. (random$ adj2 allocat$).tw.

28. single blind$.tw.

29. double blind$.tw.

30. ((treble or triple) adj blind$).tw.

31. placebo$.tw.

32. Prospective Study/

33. or/16-32

34. 10 and 15 and 33

## Observational search

**Medline**

1. exp pregnancy/

2. exp pregnancy complications/

3. exp pregnancy trimesters/

4. pregnan*.tw.

5. prenatal care/

6. postnatal care/

7. ((prenatal or pre-natal or postnatal or post-natal or antenatal or ante-natal or postpartum or post-partum) adj3 (care* or servic*)).tw.

8. (abortion* or miscarr* or trauma* or terminat* or haemorrhag* or hemorrhag*).tw.

9. ((fetal or neonatal or neo-natal) adj3 death*).tw.

10. or/1-9

11. Rh Isoimmunization/

12. "Rho(D) Immune Globulin"/ or Rh-Hr Blood-Group System/

13. ((rh or rhesus) adj4 (senti?ation* or sensibili?ation* or iso-immuni?ation* or isoimmuni?ation*)).tw.

14. (prophylax* adj5 (Rh or anti-D)).tw.

15. or/11-14

16. randomized controlled trial.pt.

17. controlled clinical trial.pt.

18. randomized.ti,ab.

19. placebo.ti,ab.

20. drug therapy.sh.

21. randomly.ti,ab.

22. groups.ti,ab.

23. or/16-22

24. 10 and 15 and 23

25. Epidemiologic studies/

26. exp case control studies/

27. exp cohort studies/

28. Case control.tw.

29. (cohort adj (study or studies)).tw.

30. Cohort analy$.tw.

31. (Follow up adj (study or studies)).tw.

32. (observational adj (study or studies)).tw.

33. Longitudinal.tw.

34. Retrospective.tw.

35. Cross sectional.tw.

36. Cross-sectional studies/

37. or/25-36

38. 10 and 15 and 37

39. Limit 38 to ed=20190901-20191130

**Embase**

1. exp pregnancy/

2. exp pregnancy complication/

3. pregnan*.tw.

4. exp prenatal care/

5. postnatal care/

6. exp postnatal care/

7. ((prenatal or pre-natal or postnatal or post-natal or antenatal or ante-natal or postpartum or post-partum) adj3 (care* or servic*)).tw.

8. (abortion* or miscarr* or trauma* or terminat* or haemorrhag* or hemorrhag*).tw.

9. ((fetal or neonatal or neo-natal) adj3 death*).tw.

10. or/1-9

11. exp rhesus isoimmunization/

12. exp rhesus D antibody/ or exp blood group rhesus system/

13. ((rh or rhesus) adj4 (senti?ation* or sensibili?ation* or iso-immuni?ation* or isoimmuni?ation*)).tw.

14. (prophylax* adj5 (Rh or anti-D)).tw.

15. or/11-14

16. clinical trial/

17. controlled clinical trial/

18. randomized controlled trial/

19. multicenter study/

20. exp randomization/

21. crossover procedure/

22. single blind procedure/

23. double blind procedure/

24. placebo/

25. randomi?ed controlled trial$.tw.

26. rct.tw.

27. (random$ adj2 allocat$).tw.

28. single blind$.tw.

29. double blind$.tw.

30. ((treble or triple) adj blind$).tw.

31. placebo$.tw.

32. Prospective Study/

33. or/16-32

34. 10 and 15 and 33

35. Clinical study/

36. Case control study/

37. Family study/

38. Longitudinal study/

39. Retrospective study/

40. Prospective study/

41. Randomized controlled trials/

42. 40 not 41

43. Cohort analysis/

44. (Cohort adj (study or studies)).mp.

45. (Case control adj (study or studies)).tw.

46. (follow up adj (study or studies)).tw.

47. (observational adj (study or studies)).tw.

48. (epidemiologic$ adj (study or studies)).tw.

49. (cross sectional adj (study or studies)).tw.

50. or/35-39,42-49

51. 10 and 15 and 50

52. Limit 51 to dd=20190901-20191130
