# Supplementary material for: Antenatal and postpartum prevention of Rh alloimmunization: A systematic review and GRADE analysis
Source: PLoS One. 2020 Sep 10;15(9):e0238844. doi: 10.1371/journal.pone.0238844 (PMC7482964; doi:10.1371/journal.pone.0238844)
Supplement: S2 File — (DOCX) [file pone.0238844.s002.docx]

# S2. Additional search details

|  | **Randomized controlled trials** | **Comparative observational studies** |
| --- | --- | --- |
| **Databases and dates** | - MEDLINE and MEDLINE in Process via OVID (1957-2019) - Embase Classic + Embase via OVID (1947-2019) - Cochrane Library including EBM Reviews - Cochrane Central Register of Controlled Trials (1991 – 2019) - EBM Reviews - Cochrane Database of Systematic Reviews (2005 – 2019) - EBM Reviews - Database of Abstracts of Reviews of Effects (1st Quarter 2016) | - MEDLINE and MEDLINE in Process via OVID (1957-2019) - Embase Classic + Embase via OVID (1947-2019) |
| **Search dates** | January 2000 to November 26, 2019 | January 2000 to November 26, 2019 |
| **Search filters** | - Embase: Scottish Intercollegiate Guidelines Network (SIGN) filter (<https://www.sign.ac.uk/search-filters.html>) - All other databases: Cochrane Highly Sensitive Search Strategy (<https://work.cochrane.org/pubmed>), sensitivity- and precision-maximizing version (2008 revision) | - An adapted version of Scottish Intercollegiate Guidelines Network (SIGN) filter (<https://www.sign.ac.uk/search-filters.html>) |
| **Grey literature (same for all study designs)** | To identify relevant literature published at any time, we searched the following websites:   - Society of Obstetricians and Gynaecologists of Canada (SOGC) - The American College of Obstetricians and Gynecologists (ACOG) - American Journal of Obstetrics and Gynecology (AJOG) - National Health Service (NHS) - National Institute for Health and Care Excellence (NICE) - Society for Maternal-Fetal Medicine (SMFM) - Royal Australian and New Zealand College of Obstetricians and Gynaecologists (RANZCOG)   Additionally, we searched:   - The bibliographies of the guidelines from the four national guideline groups: SOGC [1], ACOG [2,3], the Royal College of Obstetricians and Gynaecologists (RCOG) [4–6], RANZCOG [7], and in known systematic reviews [8–10] - Relevant systematic reviews identified through the databases searches | |

**References**

[1] Fung Kee Fung K, Eason E. Prevention of Rh Alloimmunization. SOGC Clinical Practice Guidelines. No. 133. J Obstet Gynaecol Can 2003;25:765–73.

[2] ACOG. Practice Bulletin No. 181: Prevention of Rh D Alloimmunization. Obstet Gynecol 2017;130:e57. https://doi.org/10.1097/AOG.0000000000002232.

[3] Hendrickson JE, Delaney M. Hemolytic Disease of the Fetus and Newborn: Modern Practice and Future Investigations. Transfus Med Rev 2016;30:159–64. https://doi.org/10.1016/j.tmrv.2016.05.008.

[4] RCOG. Gestational Trophoblastic Disease (Green-top Guideline No. 38) 2010.

[5] RCOG. Rhesus D Prophylaxis, The Use of Anti-D Immunoglobulin for (Green-top Guideline No. 22) 2011.

[6] RCOG. Red Cell Antibodies during Pregnancy, The Management of Women with (Green-top Guideline No. 65) 2014.

[7] RANZCOG. Guidelines for the use of Rh(D) Immunoglobulin (Anti-D) in obstetrics in Australia 2015.

[8] McBain RD, Crowther CA, Middleton P. Anti-D administration in pregnancy for preventing Rhesus alloimmunisation. Cochrane Database Syst Rev 2015:CD000020. https://doi.org/10.1002/14651858.CD000020.pub3.

[9] Crowther C, Middleton P. Anti-D administration after childbirth for preventing Rhesus alloimmunisation. Cochrane Database Syst Rev 2000:CD000021. https://doi.org/10.1002/14651858.CD000021.

[10] Karanth L, Jaafar SH, Kanagasabai S, Nair NS, Barua A. Anti-D administration after spontaneous miscarriage for preventing Rhesus alloimmunisation. Cochrane Database Syst Rev 2013:CD009617. https://doi.org/10.1002/14651858.CD009617.pub2.
