# Supplementary material for: Antenatal and postpartum prevention of Rh alloimmunization: A systematic review and GRADE analysis
Source: PLoS One. 2020 Sep 10;15(9):e0238844. doi: 10.1371/journal.pone.0238844 (PMC7482964; doi:10.1371/journal.pone.0238844)
Supplement: S3 File — (DOCX) [file pone.0238844.s003.docx]

# S3. Screening Questions

## Title and abstract screening

*1. Does this study evaluated pregnant or post-partum women who have been considered or given anti-D or Rh_o_(D) immune globulin (RhIG)?***For ref IDs 1-11999, study design must be a guideline, SR, RCT or non-RCT. For ref IDs 20000+, we are looking for comparative cohort and case-control studies.**

- Yes/unclear
- No

## Full-text screening for trials

*[1] Is this article:*

- English or French
- Other language
- Full-text not available
- Published prior to 2000

*[2] What is the study type?*

- RCT/non-RCT
- Observational
- Guideline/SR (relevant)
- Other

*[3] Does this article include Rh-negative pregnant women or women in the postpartum period (up to 42 days after childbirth)?*

- Yes
- No
- Unclear (contact author)

*[4] Is Anti-D immune globulin G compared in the two groups?*- Could be call WinRho, Rho(D), RhIg. 
- Comparisons could include: timing administered, type of administration, dosage, placebo

- Yes
- No

## Full-text screening for observational studies

*[1] Is this article:*

- English or French
- Other language
- Full-text not available
- Published prior to 2000

*[2] What is the study type?*

- Relevant guideline/SR
- RCT/non-RCT
- Observational (e.g., cohort or case-control)
- Other (e.g., narrative review, editorial, commentary)

*[3] Does this article include any of the following populations:*

- Rh-negative pregnant women or women in the postpartum period (up to 42 days after childbirth)
- Women who are not alloimmunised at the start of a prospective study
- Women who may be alloimmunised, but are evaluated retrospectively (e.g., retrospective cohort, case-control)
- Yes
- No

*[4] Does this study evaluate any of the following comparisons:*

- Anti-D compared to no anti-D (placebo) [e.g., 300 µg vs none]
- One dosage of anti-D compared to another dosage of anti-D [e.g., 300 µg vs 250 µg]
- Anti-D administration at one time compared to another time during pregnancy/childbirth [e.g., at 28 and 34 weeks vs at delivery]
- Anti-D administration through one route compared to another [e.g., IV vs intramuscular]
- Anti-D immune globulin could be call WinRho, Rho(D), RhIg.
- Yes
- No

*[5] Does this study provide results in the mother?*

- Yes
- No
- Yes, but should still be excluded _______________________
