# Supplementary material for: Antenatal and postpartum prevention of Rh alloimmunization: A systematic review and GRADE analysis
Source: PLoS One. 2020 Sep 10;15(9):e0238844. doi: 10.1371/journal.pone.0238844 (PMC7482964; doi:10.1371/journal.pone.0238844)
Supplement: S4 File — (DOCX) [file pone.0238844.s004.docx]

# S4. List of studies excluded at full-text (trials)

## Published prior to 2000

**(bolded items are included studies (or companions to included studies) captured through guideline/SR bibliography and grey lit searching, but were excluded as part of the electronic search strategy which was set to capture publication in 2000 onward)**

1. T. Anderson. Anti-D administration after childbirth for preventing Rhesus isoimmunisation. The practising midwife 1999; 2 (5): 10-11.
2. S. J. Urbaniak. The scientific basis of antenatal prophylaxis. British Journal of Obstetrics and Gynaecology, Supplement 1998; 105 (18): 11-18.
3. D. James. Anti-D prophylaxis in 1997: The Edinburgh Consensus Statement. Archives of Disease in Childhood: Fetal and Neonatal Edition 1998; 78 (3): F161-F162.
4. A. Fletcher, A. Thomson. The introduction of human monoclonal anti-D for therapeutic use. Transfusion Medicine Reviews 1995; 9 (4): 314-326.
5. M. Contreras, M. De Silva. The prevention and management of haemolytic disease of the newborn. Journal of the Royal Society of Medicine 1994; 87 (5): 256-258.
6. W. Bader, O. Behrens, W. Holle, D. H. A. Maas. The effect of antenatal rhesus prophylaxis on the antibodies. [German]. Archives of Gynecology and Obstetrics 1993; 254 (1-4): 1421-1423.
7. D. Lee. Anti-D immunoglobulin. Prescribers' Journal 1991; 31 (4): 135-137.
8. S. V. Kulkarni, S. C. Gupte, H. M. Bhatia. Efficacy of prophylactic anti-D immunoglobin injections. The Indian journal of medical research 1987; 85: 181-183.
9. C. M. Stedman, J. F. Huddleston. Causes of Rh sensitization in Alabama. The Alabama journal of medical sciences 1987; 24 (2): 140-142.
10. D. A. Verkuyl. Economics of anti-rhesus prophylaxis in an African population. The Central African journal of medicine 1987; 33 (2): 32-37.
11. W. Pollack. Rh hemolytic disease of the newborn: its cause and prevention. Progress in clinical and biological research 1981; 70: 185-302.
12. P. A. Hensleigh, W. Leslie, E. Dixon. Reduced dose of Rh(o)(D) immune globulin following induced first-trimester abortion. American Journal of Obstetrics and Gynecology 1977; 129 (4): 413-416.
13. B. Kurteva, N. Doichinova, D. Shinkov, T. Angelova, L. Ivanova. 10 years of specific prophylaxis of hemolytic disease of the newborn in Bulgaria. [Bulgarian]. Akusherstvo i ginekologiia 1979; 18 (3): 172-179.
14. E. Maroni, J. Munzinger. Postpartum rubella vaccination and anti-D prophylaxis. British medical journal 1975; 2 (5970): 541-542.
15. G. Potron, C. Quereux, C. Freal. Prevention of Rh disease by anti-D plasma. A 2-year study. [French]. Revue francaise de transfusion 1973; 16 (1): 11-31.
16. R. Cregut, M. Gosset, F. Pinon. Determination of anti-D activity in plasma pools and gamma globulins destined for Rh prevention. [French]. Revue francaise de transfusion 1973; 16 (2): 167-174.
17. P. S. Gavin. Rhesus sensitization in abortion. Obstetrics and gynecology 1972; 39 (1): 37-40.
18. J. A. Goldman, B. Eckerling. Prevention of Rh immunization following abortion. [Hebrew]. Harefuah 1972; 83 (3): 100-101.
19. W. Pollack, W. Q. Ascari, R. J. Kochesky, R. R. O'Connor, T. Y. Ho, D. Tripodi. Studies on Rh prophylaxis. 1. Relationship between doses of anti-Rh and size of antigenic stimulus. Transfusion 1971; 11 (6): 333-339.
20. **J. C. Woodrow, C. A. Clarke, R. B. McConnell, S. H. Towers, W. T. Donohoe. Prevention of Rh-haemolytic disease: results of the Liverpool "low-risk" clinical trial. British medical journal 1971; 2 (5762): 610-612.**
21. **Anonymous. Prevention of Rh-haemolytic disease: final results of the "high-risk" clinical trial. A combined study from centres in England and Baltimore. British medical journal 1971; 2 (5762): 607-609.**
22. J. Eklund, H. R. Nevanlinna. Prevention of Rh immunization in Finland. Results obtained in a two-year national anti-D programme. [Finnish]. Duodecim; laaketieteellinen aikakauskirja 1971; 87 (10): 861-866.
23. V. J. Freda, J. G. Gorman, R. S. Galen, N. Treacy. The threat of Rh immunisation from abortion. Lancet 1970; 2 (7664): 147-148.
24. **G. J. Bishop, V. I. Krieger. One millilitre injections of Rh (D) immune globulin (human) in prevention of Rh immunization. A further report on the clinical trial. The Medical journal of Australia 1969; 2 (4): 171-174.**
25. **J. C. Godel, D. I. Buchanan, J. M. Jarosch, M. McHugh. Significance of Rh-sensitization during pregnancy: its relation to a preventive programme. British medical journal 1968; 4 (5629): 479-482.**
26. N. P. Walsh, S. Peter, S. R. Hewitt. IgG anti-D in prevention of rhesus iso-immunization--a second report and review of 100 pregnancies. Journal of the Irish Medical Association 1968; 61 (375): 315-318.
27. **G. J. Bishop, V. I. Krieger, M. Tait, C. Walsh. Clinical trial of one millilitre injections of RH0 (D) immune globulin (human) in the prevention of Rh immunization: preliminary report. The Medical journal of Australia 1968; 1 (26): 1122-1127.**
28. D. V. Fairweather, D. Tacchi, A. Coxon, M. I. Hughes, S. Murray, W. Walker. Intrauterine transfusion in Rh-isoimmunization. British medical journal 1967; 4 (5573): 189-194.
29. **V. J. Freda, J. G. Gorman, W. Pollack, J. G. Robertson, E. R. Jennings, J. F. Sullivan. Prevention of Rh isoimmunization. Progress report of the clinical trial in mothers. JAMA 1967; 199 (6): 390-394.**
30. V. J. Freda, J. G. Gorman, W. Pollack. Suppression of the primary Rh immune response with passive Rh IgG immunoglobulin. The New England journal of medicine 1967; 277 (19): 1022-1023.
31. J. G. Gorman, V. J. Freda, W. J. Pollack, J. G. Robertson. Protection from immunization in Rh-incompatible pregnancies: a progress report. Bulletin of the New York Academy of Medicine 1966; 42 (6): 458-473.
32. **Anonymous. Prevention of Rh-haemolytic disease: results of the clinical trial. A combined study from centres in England and Baltimore. British medical journal 1966; 2 (5519): 907-914.**
33. C. A. Clarke, R. Finn, D. Lehane, R. B. McConnell, P. M. Sheppard, J. C. Woodrow. Dose of anti-D gamma-globulin in prevention of Rh-haemolytic disease of the newborn. British medical journal 1966; 1 (5481): 213-214.
34. V. J. Freda. Prevention of Rh disease. Haematologia 1972; 6 (1-2): 149-163.
35. J. T. Queenan, E. C. Gadow, A. C. Lopes. Role of spontaneous abortion in Rh immunization. Amer J. Obstet. Gynec. 1971; 110 (1): 128-130.
36. J. Ekllllld, H. R. Nevanlinna. Prevention of rh immunization in finland. results of a two year national anti d programme. [Finnish]. Nord.Med 1971; 85 (19): 592-596.
37. **C. A. White, R. D. Visscher, H. C. Visscher, M. E. Wade. Rho (D) immune prophylaxis. A double blind cooperative study. Obstetrics and Gynecology 1970; 36 (3): 341-346.**
38. G. J. Bishop, V. J. Krieger. Primary and secondary response in relation to the initial detection of anti Rho (D) in Rh negative mothers at risk. Med.J.Aust 1970; 57 (13): 663-668.
39. C. A. Clarke. Prevention of rhesus isoimmunization. Clinical Genetics 1970; 1 (4): 183-215.
40. C. Sprague. The role of RhoGAM in therapeutic and spontaneous abortion. Hawaii Medical Journal 1970; 29 (6): 450-451.
41. P. Borner, H. Deicher, H. H. Hoppe. Prevention of rh sensitization by i.v. administration of immunoglobulin g anti d. i. clinical results and studies of anti d dosage. [German]. Geburtsrfrauenheilk 1969; 29 (3): 203-212.
42. T. D. Stout. Prevention of rh immunization. Canadj.Rubi.Iii.Tii 1969; 60 (4): 397-405.
43. E. T. Bowe. Immunization against Rh. Postcrad 1969; Med. 45 (4): 110-114.
44. **C. De Dudok Wit, E. BorstEiiers, C. M. Van De Weerdt, G. J. Kloosterman. Prevention of rhesus immunization. a controlled clinical trial with a comparatively low dose of anti d immunoglobulin. Brit.Med.J 1968; 4 (5629): 477-479.**
45. H. Dahlstrom, H. Kjellman. Prophylaxis against rhesus immunization using immunoglobulin anti-D. [Swedish]. Lakartidningen 1968; 65 (11): 1123-1128.
46. L. N. Sussman, R. Uy, H. Berk. The prophylaxis of Rh hemolylic disease with Rh immunoglobulin. American journal of clinical pathology 1968; 50 (3): 287-290.
47. **E. R. Jennings, H. H. Dibbern, F. H. Hodell. Long Beach (California) experience with Rh immunoglobulin. Transfusion 1968; 8 (3): 146-148.**
48. **W. Pollack, J. G. German, V. J. Freda. Results of clinical trials of rhogam in women. Transf 1968; (Philad) 8 (3): 151-153.**
49. B. Chown. The suppression of rh immunization by passively administered human immunoglobulin (igg) anti-d (anti-rh). Bull Wld Hlth Org. 1967; 36 (3): 1218-1222.
50. V. J. Freda, J. G. Gorman, W. Pollack. Prevention of rh isoimmunization in obstetrics with 'rh immunoglobulin*. a progress report. J Int. Fed. Gynaec. Obstbt. 1966; 4 (3): 169-189.
51. V. J. Freda. Prevektion of rh factor isoimmunizatlon in obstetrics. preliminary results of clinical research in mothers. [Italian]. Rivista di Ostetricia e Ginecologia 1966; 21 (9): 553-565.
52. **J. C. Woodrow, C. A. Clarke, W. T. A. Donohoe, R. Finn, R. B. McConneil, P. M. Sheppard, D. Lehane, S. H. Russell, W. Kulke, C. M. Durkin. Prevention of Rh-haemolytic disease: A third report. British medical Journal (1857) 1965; 5430: 279-283.**

## Published in language other than English or French

1. E. Baiochi, L. M. Nardozza. [Alloimmunization]. Revista Brasileira de Ginecologia e Obstetricia 2009. 31 (6) 311-9-.
2. R. Corosu, R. Tillo. The role of pre-partum anti-D immunoprophylaxis: Scientific evidences. [Italian]. Giornale Italiano di Ostetricia e Ginecologia 2008. 30 (10) 295-299-.
3. T. J. Schouten, P. F. W. Strengers. Rhesus(D)immunoglobulin: Indications, availability and efficacy. The reverse side of success. [Dutch]. Pharmaceutisch Weekblad 2002. 137 (43) 1512-1516-.

## Full-text not available

1. N. C. Mashabane. Management of termination of pregnancy. Obstetrics and Gynaecology Forum 2008. 18 (2) 53-55-.
2. Anonymous. ACOG practice bulletin number 67: Medical management of abortion. Obstetrics and Gynecology 2005. 106 (4) 871-881-.

## Observational study design

1. K. Finning, P. Martin, J. Summers, E. Massey, G. Poole, G. Daniels. Effect of high throughput RHD typing of fetal DNA in maternal plasma on use of anti-RhD immunoglobulin in RhD negative pregnant women: Prospective feasibility study. Obstetrical and Gynecological Survey 2008; 63 (8): 499-500.

## Guideline/SR (relevant)

1. R. D. McBain, C. A. Crowther, P. Middleton. Anti-D administration in pregnancy for preventing Rhesus alloimmunisation. Cochrane Database of Systematic Reviews 2015; (9): CD000020.
2. L. Karanth, S. H. Jaafar, S. Kanagasabai, N. S. Nair, A. Barua. Anti-D administration after spontaneous miscarriage for preventing Rhesus alloimmunisation. Cochrane Database of Systematic Reviews 2013; (3): CD009617.
3. C. I. Okwundu, B. B. Afolabi. Intramuscular versus intravenous anti-D for preventing Rhesus alloimmunization during pregnancy. Cochrane Database of Systematic Reviews 2013; (1): CD007885.
4. O. Parant. [Comparison of the efficacy of different methods for the prevention of anti-D allo-immunization during pregnancy: targeted strategy limited to risk situations or associated with systematic prevention in the 3rd trimester]. Journal de Gynecologie, Obstetrique et Biologie de la Reproduction 2006; 35 (1 Suppl): 1S93-1S103.
5. K. Fung Kee Fung, E. Eason, J. Crane, A. Armson, S. De La Ronde, D. Farine, L. Keenan-Lindsay, L. Leduc, G. J. Reid, J. V. Aerde, R. D. Wilson, G. Davies, V. A. Desilets, A. Summers, P. Wyatt, D. C. Young, Genetics Committee Maternal-Fetal Medicine Committee. Prevention of Rh alloimmunization. Journal of Obstetrics & Gynaecology Canada 2003; 25 (9): 765-73.
6. C. Crowther, P. Middleton. Anti-D administration after childbirth for preventing Rhesus alloimmunisation. Cochrane Database of Systematic Reviews 2000; (2): CD000021.
7. K. F. K. Fung, E. Eason. No. 133-Prevention of Rh Alloimmunization. Journal of Obstetrics & Gynaecology Canada: JOGC 2018; 40 (1): e1-e10.
8. H. Pilgrim, M. Lloyd-Jones, A. Rees. Routine antenatal anti-D prophylaxis for RhD-negative women: A systematic review and economic evaluation. Health Technology Assessment 2009; 13 (10): iii-87.
9. B. A. Murphy, A. R. Hansen, J. M. Howell, B. Simmons, S. V. Cantrill, W. C. Dalsey, A. S. Jagoda, S. A. Colucciello, W. W. Decker, F. M. Fesmire, S. A. Godwin, J. S. Huff, A. H. Itzkowitz, S. Karas Jr, E. K. Kuffner, T. W. Lukens, B. E. Marett, T. P. Martin, J. Moore, D. L. Morgan, D. Nazarian, S. M. Silvers, E. P. Sloan, S. Wall, R. L. Wears, S. J. Wolf. Clinical policy: Critical issues in the initial evaluation and management of patients presenting to the emergency department in early pregnancy. Annals of Emergency Medicine 2003; 41 (1): 123-133.
10. American College of Obstetricians and Gynecologists. Practice Bulletin No. 181: Prevention of Rh D Alloimmunization. Obstet Gynecol. 2017; 130 (2): e57-.
11. Qureshi H, Massey E, Kirwan D, Davies T, Robson S, White J, Jones J, Allard S. BCSH guideline for the use of anti-D immunoglobulin for the prevention of haemolytic disease of the fetus and newborn. Transfusion Medicine 2014; 24: 8-20.
12. Royal College of Obstetricians and Gynaecologists. Red Cell Antibodies during Pregnancy, The Management of Women with (Green-top Guideline No. 65). 2014.
13. RANZCOG. Guidelines for the use of Rh(D) Immunoglobulin (Anti-D) in obstetrics in Australia. 2015.
14. New south Wales Government. Maternity - Rh (D) Immunoglobulin (Anti D). 2015 Sep.
15. Vain J, Chari R, Maslovitz S, Farine D. Guidelines for the Management of a Pregnant Trauma Patient. J Obstet Gynaecol Can 2015; 37 (6): 553–571.
16. American College of Obstetricians and Gynecologists. Management of Alloimmunization During Pregnancy. Obstetrics & Gynecology 2018 Mar; 131 (3): e82-e90.
17. National Institute For Health Care Excellence. Routine antenatal anti-D prophylaxis for women who are rhesus D negative. 2008 Aug.
18. Royal College of Obsetricians and Gyneacologists. The Use of Anti-D Immunoglobulin for Rhesus D Prophylaxis. Green-top Guideline No. 22. 2011 Mar.

## Other study design

1. J. D. Sperling, J. D. Dahlke, D. Sutton, J. M. Gonzalez, S. P. Chauhan. Prevention of RhD Alloimmunization: A Comparison of Four National Guidelines. American Journal of Perinatology 2018; 35 (2): 110-119.
2. B. Branger, N. Winer. [Epidemiology of anti-D allo-immunization during pregnancy]. Journal de Gynecologie, Obstetrique et Biologie de la Reproduction 2006; 35 (1 Suppl): 1S87-1S92.
3. E. Lopriore. Neonatal management and outcome in hemolyic disease of the fetus and newborn. Vox sanguinis 2017; 112 (10).
4. C. E. Pennell, J. C. Cheng, B. Penova-Veselinovic, C. A. Wang, B. Ingleby, C. C. Arnold, A. L. Barr, M. K. White, S. W. White. Single dose anti-D prophylaxis in pregnancy: is it time to change? Reproductive sciences 2017. Conference: 64th annual scientific meeting of the society for gynecologic investigation, SGI. 2017. United states 24 (1 Supplement 1): 117A-.
5. C. Denison, L. Parry, P. Hb Bolton-Maggs. Errors related to anti-D immunoglobulin (Ig) in the 2016 SHOT report-are mothers and babies still at risk? Transfusion Medicine 2017; 27 (Supplement 2): 63-.
6. T. Powley, J. Thyer, A. Gould, C. Hyland, J. Wong. Fifty years of RhD immunoglobulin therapy in Australia. Vox Sanguinis 2017; 112 (Supplement 1): 113-.
7. C. P. Shao. Transfusion of RhD-positive blood in "Asia type" DEL recipients. New England Journal of Medicine 2010; 362 (5): 472-473.
8. G. M. Liumbruno, A. D'Alessandro, F. Rea, V. Piccinini, L. Catalano, G. Calizzani, S. Pupella, G. Grazzini. The role of antenatal immunoprophylaxis in the prevention of maternal-foetal anti-Rh(D) alloimmunisation. Blood Transfusion 2010; 8 (1): 8-16.
9. L. W. Chia, J. Davies, R. Chant, S. Simpson. Routine antenatal anti-D prophylaxis (RAADP) in RhD negative women. What is the best approach? British Journal of Haematology 2009; 1: 78-79.
10. K. J. Moise Jr. Management of rhesus alloimmunization in pregnancy. Obstetrics and Gynecology 2008; 112 (1): 164-176.
11. J. Bowman. Rh-immunoglobulin: Rh prophylaxis. Best Practice and Research: Clinical Haematology 2006; 19 (1): 27-34.
12. K. S. Ferentz, L. S. Nesbitt. Common Problems and Emergencies in the Obstetric Patient. Primary Care - Clinics in Office Practice 2006; 33 (3): 727-750.
13. M. C. Fox, M. D. Creinin. Modern management of first trimester miscarriage. Contemporary Clinical Gynecology and Obstetrics 2002; 2 (1): 47-58.
14. Hendrickson JE, Delaney M. Hemolytic Disease of the Fetus and Newborn: Modern Practice and Future Investigations. Transfus Med Rev 2016; 4 (30): 159–164.
15. Royal College of Obstetricians and Gynaecologists. Gestational Trophoblastic Disease (Green-top Guideline No. 38). 2010.
16. Kent J, Farrell A-M, Soothill P. Routine administration of Anti-D: the ethical case for offering pregnant women fetal RHD genotyping and a review of policy and practice. BMC Pregnancy and Childbirth 2014; 14: 87-.
17. Bhutani VK, Zipursky A, Blencowe H, Khanna R, Sgro M, Ebbesen F, Bell J, Mori R, Slusher TM, Fahmy N, Paul VK, Du L, Okolo AA, de Almeida M-F, Olusanya BO, Kumar P, Cousens S, Lawn JE. Neonatal hyperbilirubinemia and Rhesus disease for the newborn: incidence and impairment estimates for 2010 at regional and global levels. Pediatric Research 2013 Dec; 74 (s1).
18. Society for Maternal-Fetal Medicine, Mari G, Norton ME, Stone J, Berghella V, Sciscione AC, Tate D, Schenone MH. Society for Maternal-Fetal Medicine (SMFM) Clinical Guideline #8: The fetus at risk for anemia - diagnosis and management. American Journal of Obstetrics & Gynecology 2015 Jun: 697-710.
19. Sandler GS, Flegel WA, Westhoff CM, Denomme GA, Delaney M, Keller MA, Johnson ST, Katz L, Queenan JT, Vassallo RR, Simon CD. It's time to phase-in RHD genotyping for patients with a serological weak D phenotype. Transfusion 2015 Mar; 55 (3): 680-689.

## Anti-D immune globulin G not compared

1. M. Darlington, B. Carbonne, A. Mailloux, Y. Brossard, A. Levy-Mozziconacci, A. Cortey, H. Maoulida, T. Simon, A. Rousseau, I. Durand-Zaleski, Geniferh Study Group. Effectiveness and costs of non-invasive foetal RHD genotyping in rhesus-D negative mothers: a French multicentric two-arm study of 850 women. BMC Pregnancy & Childbirth 2018; 18 (1): 496.
