# Supplementary material for: Antenatal and postpartum prevention of Rh alloimmunization: A systematic review and GRADE analysis
Source: PLoS One. 2020 Sep 10;15(9):e0238844. doi: 10.1371/journal.pone.0238844 (PMC7482964; doi:10.1371/journal.pone.0238844)
Supplement: S5 File — (DOCX) [file pone.0238844.s005.docx]

# S5. List of studies excluded at full text from observational study search

## Other language

1. L. Messeter. [Rh-prophylaxis or not? Obstetric departments should make decisions if there are medical reasons which justify the treatment]. Lakartidningen 2000; 97(51-52):6030-3.
2. G. Gessoni, S. Valverde, A. Giacomini, F. Antico, M. Salvadego, N. Arreghini, G. De Fusco, M. Fezzi, G. Marchiori, F. Manoni. [Foetal-maternal alloimmunizations in the South-East area of the Venice province]. Minerva Ginecologica 2002; 54(4):333-8.
3. M. A. Cianciarullo, M. E. Ceccon, F. A. Vaz. Prevalence of immunohematologic tests at birth and the incidence of hemolytic disease in the newborn. [Portuguese]. Revista da Associacao Medica Brasileira (1992) 2003; 49(1):45-53.
4. E. Hernandez-Andrade, J. R. Ahued-Ahued. [Transvaginal bleeding in pregnancy, as risk factor of Rhesus-D antigen isoimmunization]. Salud Publica de Mexico 2003; 45(6):492-6.
5. H. A. Baptista Gonzalez, F. Rosenfeld Mann. [Prevention of maternal isoimmunization to RhD antigen]. Salud Publica de Mexico 2004; 46(3):194-5; author reply 196-7.
6. N. Bujandric, M. K. Milanovic. [The importance of immunohematology testing in the neonatal period]. Medicinski Pregled 2013; 66(7-8):317-21.

## Full-text not available

1. F. Santos, A. Ferreira, M. Bini Antunes, M. Amil. Maternal alloantibodies and development of hemolytic disease of fetus and newborn: A study from a single center. Blood Transfusion 2018; 16 (Supplement 1):s87-s88.
2. S. N. Kapadia, K. R. Bansal, K. Parmar, H. Panot, F. Bandi. Assessment of fetomaternal hemorrhage in rhesus D-negative postpartum women by kleihauer-betke test. Journal of SAFOG 2016; 8(3):181-184.

## Relevant guideline/SR

1. J. Chilcott, M. Lloyd Jones, J. Wight, K. Forman, J. Wray, C. Beverley, P. Tappenden. A review of the clinical effectiveness and cost-effectiveness of routine anti-D prophylaxis for pregnant women who are rhesus-negative. Health Technology Assessment 2003; 7(4).
2. Acep Clinical Policies Committee, Physicians Clinical Policies Subcommittee on Early Pregnancy. American College of Emergency. Clinical policy: critical issues in the initial evaluation and management of patients presenting to the emergency department in early pregnancy. Annals of Emergency Medicine 2003; 41(1):123-33.

## Other study type (e.g., narrative review, editorial)

1. R. Aggarwal, R. Seth, V. K. Paul, A. K. Deorari. High dose intravenous immunoglobulin therapy in the treatment of rhesus hemolytic disease. Journal of Tropical Pediatrics 2002; 48(2):116-117.
2. A. R. Chauhan, M. S. Bhattacharyya, N. Turakhia, G. V. Daftary. Efficacy and safety of monoclonal anti-D immunoglobulin in comparison with polyclonal anti-D immunoglobulin in prevention of rho isoimmunization. Journal of the Association of Physicians of India 2002; 50:1341-2.
3. G. Lobato, C. S. Soncini. RhD prophylaxis failure in Rio de Janeiro, Brazil. International Journal of Gynaecology & Obstetrics 2008; 100(3):276-7.
4. N. Kadwadkar, J. Lo. To review practice in the management of rhesus D-negative women during pregnancy. Archives of Disease in Childhood: Fetal and Neonatal Edition 2010; 1):Fa52-Fa53.
5. A. D. Kuruppu. Current status and future goals of Antenatal antibody screening in Sri Lanka. Vox Sanguinis 2010; 1):402.
6. A. Kulic, V. Libek, A. Strugar, I. Simic, N. Rankovic. Differences in RBC alloimmunization between pregnant woman and patiens in CHC zemun. Vox Sanguinis 2011; 1):252.
7. T. Prior, C. Johnstone-Ayliffe, C. Ong, F. Regan, S. Kumar. Procedure related complications of fetal blood sampling and in-utero transfusion. Archives of Disease in Childhood: Fetal and Neonatal Edition 2011; 1):Fa63.
8. A. Sange, V. Skelton. Does the use of cell salvage in Rhesus D antigen negative women cause alloimmunisation? International Journal of Obstetric Anesthesia 2011; 1):S16.
9. M. Patwardhan, M. Allan, N. Ramskill. An audit of the ectopic pregnancy pathway at a District General Hospital. BJOG: An International Journal of Obstetrics and Gynaecology 2012; 1):213-214.
10. E. Tiblad. First trimester non-invasive screening for fetal RHD and targeted antenatal anti-D prophylaxis - Does it work? Acta Obstetricia et Gynecologica Scandinavica 2012; 159):53.
11. E. Tiblad, A. T. Wikman, E. Nordlander, G. Ajne, A. Karlsson, A. B. Olerup, M. Westgren. First trimester non-invasive screening for fetal RHD and targeted antenatal anti-D prophylaxis. Prenatal Diagnosis 2012; 1):29.
12. A. Verma, V. Kumar, M. Pradhan. Red cell alloimmunization in pregnant women: An experience from a tertiary care teaching hospital of North India. Transfusion 2012; 3):140A-141A.
13. A. Amirthanayagam, F. Regan. Why is anti-D sensitisation still happening in women of child-bearing age? Transfusion Medicine 2013; 2):60-61.
14. P. L. Tazzari, F. Ricci, S. Manfroi, P. Pagliaro. Experience in the evaluation of foeto-maternal haemorrhage by flow cytometry. Blood Transfusion 2013; 11(3):462-3.
15. M. Adachi, A. Takeshita, D. W. Kim, K. S. Han, S. Y. Kwon, H. O. Kim, J. S. Suh, H. Watanabe, M. Uchikawa, S. Kino, H. Ohto. Alloimmunity to erythrocytes in patients during pregnancy in South Korea and JAPAN; recent results from a cooperative international study of alloimmunity to antigen diversity in asian populations. Blood. Conference: 56th Annual Meeting of the American Society of Hematology, ASH 2014; 124(21).
16. H. Arentz-Hansen, K. G. Brurberg, M. K. Kvamme, A. Stoinska-Schneider, B. Hofmann, S. S. Ormstad, B. Fure. . Knowledge Centre for the Health Services at The Norwegian Institute of Public Health (NIPH) 2014; NIPH Systematic Reviews:Executive Summaries.
17. T. C. Stegmann, S. Q. Nagelkerke, D. Van Winkelhorst, T. W. Kuijpers, G. Vidarsson, C. E. Van Der Schoot. RhD immunization despite adequate immunoprophylaxis: Role of Fc gamma receptor gene polymorphisms. Blood. Conference: 56th Annual Meeting of the American Society of Hematology, ASH 2014; 124(21):.
18. A. J. Keidan, T. Davies, P. Bolton-Maggs, D. Poles. Anti-D immunisation in pregnancy - An ongoing study from the Serious Hazards of Transfusion UK haemovigilance scheme (SHOT). Vox Sanguinis 2015; 1):328.
19. J. M. Sgro, R. Ombao, M. Anderson, T. Jegathesan, E. Krok, J. Baker, D. M. Campbell, K. Pavenski. Prevalence of clinically significant prenatal red blood cell alloantibodies. Transfusion 2016; 56 (Supplement 4):147A.
20. E. Lopriore. Neonatal management and outcome in hemolyic disease of the fetus and newborn. Vox Sanguinis 2017; 112 (Supplement 1):10.
21. E. Muniz-Diaz. Haemolytic disease of the foetus and the newborn (HDFN): The laboratory perspective. Vox Sanguinis 2017; 112 (Supplement 1):9.
22. T. C. Stegmann, B. Veldhuisen, S. Q. Nagelkerke, D. Winkelhorst, H. Schonewille, E. P. Verduin, T. W. Kuijpers, M. de Haas, G. Vidarsson, C. E. van der Schoot. RhIg-prophylaxis is not influenced by FCGR2/3 polymorphisms involved in red blood cell clearance. Blood 2017; 129(8):1045-1048.
23. K. Telfer. A review of anti-D prophylaxis in Tayside-identifying reasons for sensitisation despite maternal participation in the routine anti-D prophylaxis programme. Transfusion Medicine 2017; 27 (Supplement 2):51-52.
24. C. Zwiers, L. Vermij, J. Koelewijn, D. Oepkes, M. De Haas, E. Van Der Schoot. Rh-immunoprophylaxis and ABO incompatibility protect against non-RHD alloimmunization by pregnancy. Vox Sanguinis 2017; 112 (Supplement 1):69-70.
25. J. Keidan, D. Poles, P. Bolton-Maggs. Anti-D immunisation in pregnancy: Cases reported to SHOT 2012 to 2017. Do obesity and prolonged gestation pose increased risks? Transfusion Medicine 2018; 28 (Supplement 1):51.
26. C. Lyon, A. English. PURL: A new protocol for RhD-negative pregnant women? Journal of Family Practice 2018; 67(5):306;308;319.
27. Hartwell EA. Use of Rh immune globulin: ASCP Practice Parameter. American Society of Clinical Pathologists. Am J Clin Pathol 1998; 110:281–292.
28. Urbaniak S. The scientific basis of antenatal prophylaxis. Br J Obstet Gynaecol 1998; 105:11-18.
29. Turner RM, Lloyd-Jones M, Anumba DO, Smith GC, Spiegelhalter DJ, Squires H, et al. Routine antenatal anti-D prophylaxis in women who are Rh(D) negative: meta-analyses adjusted for differences in study design and quality. PLoS One 2012; 7:e30711.
30. Robson S, Lee D, Urbaniak S. Anti-D immunoglobulin in RhD prophylaxis. Br J Obstet Gynaecol 1998; 105:129-134.

## Does not evaluate a relevant population

1. Y. C. Cheong, J. Goodrick, P. M. Kyle, P. Soothill. Management of anti-Rhesus-D antibodies in pregnancy: a review from 1994 to 1998. Fetal Diagnosis & Therapy 2001; 16(5):294-8.
2. C. Frohn, L. Dumbgen, J. M. Brand, S. Gorg, J. Luhm, H. Kirchner. Probability of anti-D development in D- patients receiving D+ RBCs. Transfusion 2003; 43(7):893-898.
3. Bowman JM, Pollock JM, Penston LE. Fetomaternal transplacental hemorrhage during pregnancy and after delivery. Vox Sang 1986; 51:117–121.

## Does not include a comparison of interest

1. J. Morrison. Audit of anti-D immunoglobulin administration to pregnant Rhesus D negative women following sensitising events. Journal of Obstetrics & Gynaecology 2000; 20(4):371-3.
2. S. Vause, J. Wray, C. Bailie. Management of women who are Rhesus D negative in Northern Ireland. Journal of Obstetrics and Gynaecology 2000; 20(4):374-377.
3. I. Rennie, A. Smith, R. Smith, P. S. Rawlinson, P. Clark. An audit of the investigation and use of anti-D immunoglobulin prophylaxis in Tayside. Health Bulletin 2001; 59(3):150-4.
4. L. Weinberg. Use of anti-D immunoglobulin in the treatment of threatened miscarriage in the accident and emergency department. Emergency Medicine Journal 2001; 18(6):444-7.
5. K. D. Balderston, C. V. Towers, P. J. Rumney, D. Montgomery. Is the incidence of fetal-to-maternal hemorrhage increased in patients with third-trimester bleeding? American Journal of Obstetrics & Gynecology 2003; 188(6):1615-8; discussion 1618-21.
6. M. David, J. Smidt, F. C. K. Chen, U. Stein, J. W. Dudenhausen. Risk factors for fetal-to-maternal transfusion in Rh D-negative women - Results of a prospective study on 942 pregnant women. Journal of Perinatal Medicine 2004; 32(3):254-257.
7. J. F. Tuohy, M. Sangalli, L. Kim. Red blood cell iso-immunisation in the Wellington area of New Zealand: the case for antenatal prophylaxis. Australian & New Zealand Journal of Obstetrics & Gynaecology 2004; 44(5):458-9.
8. B. M. Augustson, E. A. Fong, D. E. Grey, J. I. Davies, W. N. Erber. Postpartum anti-D: can we safely reduce the dose? Medical Journal of Australia 2006; 184(12):611-3.
9. J. F. Dricot, J. M. Minon, J. P. Schaaps, P. Dewez, J. M. Foidart. [Fetal RHD in maternal plasma in prenatal follow-up]. Revue Medicale de Liege 2006; 61(12):820-6.
10. I. Z. MacKenzie, F. Roseman, J. Findlay, K. Thompson, E. Jackson, J. Scott, M. Reed. The kinetics of routine antenatal prophylactic intramuscular injections of polyclonal anti-D immunoglobulin. BJOG: An International Journal of Obstetrics & Gynaecology 2006; 113(1):97-101.
11. B. Chaffe, J. Ford, V. Bills. Routine antenatal anti-D prophylaxis and patient compliance with the two-dose regimen. Transfusion Medicine 2007; 17(5):399-403.
12. A. Puangsricharern, S. Suksawat. Prevalence of Rh negative pregnant women who attended the antenatal clinic and delivered in Rajavithi Hospital: 2000-2005. Journal of the Medical Association of Thailand 2007; 90(8):1491-4.
13. G. Ben-David, E. Sheiner, A. Levy, O. Erez, M. Mazor. An increased risk for non allo-immunization related intrauterine fetal death in RhD-negative patients. Journal of Maternal-Fetal & Neonatal Medicine 2008; 21(4):255-9.
14. T. Gottvall, D. Filbey. Alloimmunization in pregnancy during the years 1992-2005 in the central west region of Sweden. Acta Obstetricia et Gynecologica Scandinavica 2008; 87(8):843-8.
15. I. Z. Mackenzie, F. Roseman, J. Findlay, K. Thompson, K. McPherson. Clinical validation of routine antenatal anti-D prophylaxis questions the modelling predictions adopted by NICE for Rhesus D sensitisation rates: results of a longitudinal study. European Journal of Obstetrics, Gynecology, & Reproductive Biology 2008; 139(1):38-42.
16. S. Boulet, C. Krause, H. Tixier, M. Bardou, P. Sagot. Relevance of new recommendations on routine antenatal prevention of rhesus immunization: an appraisal based on a retrospective analysis of all cases observed in two French administrative areas of 3 million inhabitants. European Journal of Obstetrics, Gynecology, & Reproductive Biology 2009; 146(1):65-70.
17. J. M. Koelewijn, T. G. Vrijkotte, M. de Haas, C. E. van der Schoot, G. J. Bonsel. Risk factors for the presence of non-rhesus D red blood cell antibodies in pregnancy. BJOG: An International Journal of Obstetrics & Gynaecology 2009; 116(5):655-64.
18. L. Leyenaar, V. M. Allen, H. E. Robinson, M. Parsons, M. C. Van den Hof. Peripartum factors predicting the need for increased doses of postpartum rhesus immune globulin. Journal of Obstetrics & Gynaecology Canada: JOGC 2010; 32(8):739-44.
19. C. P. Shao, H. Xu, Q. Xu, G. D. Sun, J. P. Li, B. W. Zhang, X. H. Liang, Z. Liu, Y. Zhou, D. Li, N. B. Zhuang. Antenatal Rh prophylaxis is unnecessary for "Asia type" DEL women. Transfusion Clinique et Biologique 2010; 17(4):260-4.
20. N. S. Bondagji. Rhesus alloimmunization in pregnancy. A tertiary care center experience in the Western region of Saudi Arabia. Saudi Medical Journal 2011; 32(10):1039-45.
21. S. Dajak, V. Stefanovic, V. Capkun. Severe hemolytic disease of fetus and newborn caused by red blood cell antibodies undetected at first-trimester screening (CME). Transfusion 2011; 51(7):1380-8.
22. F. Mol, E. van den Boogaard, N. M. van Mello, F. van der Veen, B. W. Mol, W. M. Ankum, P. van Zonneveld, A. B. Dijkman, H. R. Verhoeve, A. Mozes, M. Goddijn, P. J. Hajenius. Guideline adherence in ectopic pregnancy management. Human Reproduction 2011; 26(2):307-15.
23. R. T. Griffey, B. C. Chen, N. W. Krehbiel. Performance in appropriate Rh testing and treatment with Rh immunoglobulin in the emergency department. Annals of Emergency Medicine 2012; 59(4):285-93.
24. L. Koby, A. Grunbaum, A. Benjamin, R. Koby, H. A. Abenhaim. Anti-D in Rh(D)-negative pregnant women: are at-risk pregnancies and deliveries receiving appropriate prophylaxis? Journal of Obstetrics & Gynaecology Canada: JOGC 2012; 34(5):429-435.
25. M. Lubusky, O. Simetka, M. Studnickova, M. Prochazka, M. Ordeltova, K. Vomackova. Fetomaternal hemorrhage in normal vaginal delivery and in delivery by cesarean section. Transfusion 2012; 52(9):1977-1982.
26. T. Rekik, I. Ben Amor, N. Louati, H. Rekik, H. Menif, J. Gargouri. [Irregular antibody testing during pregnancy in Tunisia: clinical study of 5369 women]. Transfusion Clinique et Biologique 2012; 19(2):64-73.
27. E. Tiblad, A. Wikman, A. Rane, Y. Jansson, M. Westgren. Pharmacokinetics of 250 mug anti-D IgG in the third trimester of pregnancy: an observational study. Acta Obstetricia et Gynecologica Scandinavica 2012; 91(5):587-92.
28. N. Altuntas, I. Yenicesu, O. Himmetoglu, F. Kulali, E. Kazanci, S. Unal, S. Aktas, I. Hirfanoglu, E. Onal, C. Turkyilmaz, E. Ergenekon, E. Koc, Y. Atalay. The risk assessment study for hemolytic disease of the fetus and newborn in a University Hospital in Turkey. Transfusion & Apheresis Science 2013; 48(3):377-80.
29. P. H. Bolton-Maggs, T. Davies, D. Poles, H. Cohen. Errors in anti-D immunoglobulin administration: retrospective analysis of 15 years of reports to the UK confidential haemovigilance scheme. BJOG: An International Journal of Obstetrics & Gynaecology 2013; 120(7):873-8.
30. R. M. Kaufman, K. S. Schlumpf, D. J. Wright, D. J. Triulzi, II Nhlbi Retrovirus Epidemiology Donor Study. Does Rh immune globulin suppress HLA sensitization in pregnancy? Transfusion 2013; 53(9):2069-77.
31. E. Tiblad, M. Westgren, D. Pasupathy, A. Karlsson, A. T. Wikman. Consequences of being Rhesus D immunized during pregnancy and how to optimize new prevention strategies. Acta Obstetricia et Gynecologica Scandinavica 2013; 92(9):1079-85.
32. S. Dajak, D. Roje, Z. H. Haspl, P. E. Magliae. The importance of antenatal prevention of RhD immunisation in the first pregnancy. Blood Transfusion 2014; 12(3):410-415.
33. S. Chacham, D. S. Reddy, U. N. Reddy, W. Khan, S. Nandita, S. Anumula, J. Sravani, F. Adil. Neonatal outcomes of rh-negative pregnancies in a tertiary level neonatal intensive care unit: A prospective study. Journal of Comprehensive Pediatrics 2016; 7 (3).
34. S. Fahim, M. Sarosh,Nikhat. Immunoprophylaxis in RH incompatability. Rawal Medical Journal 2016; 41(3):339-341.
35. J. L. Hannon, G. Clarke. Laboratory management of perinatal patients with apparently "new" anti-D. Immunohematology 2016; 32(3):108-111.
36. J. Lukacevic Krstic, S. Dajak, J. Bingulac-Popovic, V. Dogic, J. Mratinovic-Mikulandra. Anti-D Antibodies in Pregnant D Variant Antigen Carriers Initially Typed as RhD+. Transfusion Medicine and Hemotherapy 2016; 43(6):419-424.
37. M. Jerkovic Raguz, D. Sumanovic Glamuzina, J. Brzica, T. Gruica. The Incidence and Effects of Alloimmunization in Pregnancy during the Period 2000-2013. Geburtshilfe und Frauenheilkunde 2017; 77(7):780-785.
38. I. Ksibi, R. Achour, W. Bel Haj Ammar, M. Cheour, M. Ben Amara, K. Neji, S. Kacem. [Anti-D prophylaxis in fetal-maternal erythrocyte incompatibility in Tunisia]. Archives de Pediatrie 2017; 24(10):942-949.
39. C. J. McCauley, K. Morris, K. Maguire. A review of maternal alloimmunisation to Rh D in Northern Ireland. Transfusion Medicine 2017; 27(2):132-135.
40. M. Darlington, B. Carbonne, A. Mailloux, Y. Brossard, A. Levy-Mozziconacci, A. Cortey, et al. Effectiveness and costs of non-invasive foetal RHD genotyping in rhesus-D negative mothers: A French multicentric two-arm study of 850 women. BMC Pregnancy and Childbirth 2018; 18 (1).
41. C. Zwiers, J. M. Koelewijn, L. Vermij, J. van Sambeeck, D. Oepkes, M. de Haas, C. E. van der Schoot. ABO incompatibility and RhIG immunoprophylaxis protect against non-D alloimmunization by pregnancy. Transfusion 2018; 58(7):1611-1617.
42. S Mayne, JH Parker, TA Harden, SD Dodds, JA Beale. Rate of RhD sensitisation before and after implementation of a community based antenatal prophylaxis programme.. BMJ (Clinical research ed.) 1997; 315(7122).
43. Howard HL, Martlew VJ, McFadyen IR, Clarke CA. Preventing Rhesus D haemolytic disease of the newborn by giving anti-D immunoglobulin: are the guidelines being adequately followed? Br J Obstet Gynaecol 1997; 104:37-41.
44. Hermann M, Kjellman H, Ljunggren C. Antental prophylaxis of Rh immunization with 250 micrograms anti-D. *Acta Obstetricia et Gynecologica Scandivavica* 1984; 124: 1-15.

## Does not provide results for the woman

1. Maayan-Metzger, T. Schwartz, J. Sulkes, P. Merlob. Maternal anti-D prophylaxis during pregnancy does not cause neonatal haemolysis. Archives of Disease in Childhood Fetal & Neonatal Edition 2001; 84(1):F60-2.

## Other reasons for exclusion

**Provides only a case series for those who were immunized. No information on the women who were not immunized.**

1. J. M. Koelewijn, M. de Haas, T. G. Vrijkotte, G. J. Bonsel, C. E. van der Schoot. One single dose of 200 microg of antenatal RhIG halves the risk of anti-D immunization and hemolytic disease of the fetus and newborn in the next pregnancy. Transfusion 2008; 48(8):1721-9.

**All cases were given the same dose, it is unclear what the controls were given, but the results are presented to look at risk factors of failure**

1. J. M. Koelewijn, M. de Haas, T. G. Vrijkotte, C. E. van der Schoot, G. J. Bonsel. Risk factors for RhD immunisation despite antenatal and postnatal anti-D prophylaxis. BJOG: An International Journal of Obstetrics & Gynaecology 2009; 116(10):1307-14.

**The intent of the study was to evaluate compliance between the two protocols, and not look at the effectiveness of each protocol**

1. Z. MacKenzie, S. Dutton, F. Roseman. Evidence to support the single-dose over the two-dose protocol for routine antenatal anti-D Rhesus prophylaxis: a prospective observational study. European Journal of Obstetrics, Gynecology, & Reproductive Biology 2011; 158(1):42-6.

**Retrospective case-series, looking at cases and whether or not they were given immunoprophylaxis**

1. K. G. Badami, J. Parker, A. Kenny, S. Warrington. Incidence of maternal sensitisation to Rh(D) in Christchurch, New Zealand and reasons for prophylaxis failures. New Zealand Medical Journal 2014; 127(1388):40-6.

**Mentioned in discussion of another study, with no author contact information and information comes from unpublished work**

1. M. Hermann, H. Kjellman, C. Ljunggren. Postnatal Rh-prophylaxis with immunoglobulin anti-D. A clinical study at Vaxjo Hospital, Sweden 1968-1977. Data on file, 1979.
