# Supplementary material for: Antenatal and postpartum prevention of Rh alloimmunization: A systematic review and GRADE analysis
Source: PLoS One. 2020 Sep 10;15(9):e0238844. doi: 10.1371/journal.pone.0238844 (PMC7482964; doi:10.1371/journal.pone.0238844)
Supplement: S6 File — (DOCX) [file pone.0238844.s006.docx]

**S6. Results tables, GRADE, and Forest Plots**

Contents

[1. Postpartum administration 3](#_Toc36199068)

[1.1 Postpartum RhIg compared to No postpartum treatment (or placebo) 3](#_Toc36199069)

[1.1.1. Results table 3](#_Toc36199070)

[1.1.2. GRADE 4](#_Toc36199071)

[1.1.3. Forest plot for Rh alloimmunisation at 6 months postpartum (any dose) 9](#_Toc36199072)

[1.1.4. Forest plot for Rh alloimmunisation at 6 months postpartum (by dose) 10](#_Toc36199073)

[1.1.5. Forest plot for Rh alloimmunisation at subsequent Rh-positive pregnancy (any dose) 11](#_Toc36199074)

[1.1.6. Forest plot for Rh alloimmunisation at subsequent Rh-positive pregnancy (by dose) 12](#_Toc36199075)

[1.1.7. Forest plot for sensitisation in second pregnancy (observational data) 13](#_Toc36199076)

[1.2 Higher-dose postnatal RhIg compared to Lower-dose postnatal RhIg 14](#_Toc36199077)

[1.2.1. Results table 14](#_Toc36199078)

[1.2.2. GRADE 15](#_Toc36199079)

[1.2.3. Forest plot for Rh alloimmunisation at 6 months postpartum 19](#_Toc36199080)

[1.2.4. Forest plot for Rh alloimmunisation at end of second d-positive pregnancy 20](#_Toc36199081)

[2. Antenatal administration 21](#_Toc36199082)

[2.1 Antenatal RhIg (any dose) vs No antenatal RhIg 21](#_Toc36199083)

[2.1.1. Results table 21](#_Toc36199084)

[2.1.2. GRADE 22](#_Toc36199085)

[2.1.3. Forest plot for Rh alloimmunisation (among those with Rh-positive infants) 26](#_Toc36199086)

[2.2 Antenatal plus postnatal RhIg vs No RhIg 27](#_Toc36199087)

[2.2.1. Results table 27](#_Toc36199088)

[2.2.2 GRADE 28](#_Toc36199089)

[2.2.3. Forest plot for sensitisation in second pregnancy 30](#_Toc36199090)

[2.3 Two-dose antenatal RhIg vs One-dose antenatal RhIg 31](#_Toc36199091)

[2.3.1. Results table 31](#_Toc36199092)

[2.4 Two-dose antenatal plus postnatal RhIg vs One-dose antenatal plus postnatal RhIg 32](#_Toc36199093)

[2.4.1. Results table 32](#_Toc36199094)

[2.4.2. GRADE 33](#_Toc36199095)

[2.5 Antenatal plus postnatal RhIg vs Postnatal RhIg 35](#_Toc36199096)

[2.5.1. Results table 35](#_Toc36199097)

[2.5.2. GRADE 36](#_Toc36199098)

[2.5.3. Forest plot for Rh alloimmunisation postpartum or in second pregnancy 38](#_Toc36199099)

[2.6 Shorter interval vs Longer interval between RhIg and delivery 39](#_Toc36199100)

[2.6.1. Results table 39](#_Toc36199101)

[2.6.2. GRADE 40](#_Toc36199102)

[2.6.3. Forest plot for Rh alloimmunisation six months postpartum 42](#_Toc36199103)

[3. Abortion 43](#_Toc36199104)

[3.1 RhIg vs No RhIg (placebo) given after spontaneous abortion 43](#_Toc36199105)

[3.1.1. Results table 43](#_Toc36199106)

[3.1.2. GRADE 44](#_Toc36199107)

[3.2 Higher-dose vs Reduced-dose RhIg following first trimester vacuum abortion 46](#_Toc36199108)

[3.2.1. Results table 46](#_Toc36199109)

[3.2.2. GRADE 47](#_Toc36199110)

[4. Amniocentesis 49](#_Toc36199111)

[4.1 RhIg vs No RhIg after amniocentesis 49](#_Toc36199112)

[4.1.1. Results table 49](#_Toc36199113)

[4.1.2. GRADE 50](#_Toc36199114)

[4.1.3 Forest plot for Rh immunisation from most recent pregnancy 52](#_Toc36199115)

[5. Intramuscular vs Intravenous 53](#_Toc36199116)

[5.1. Antenatal RhIg given intramuscularly vs Antenatal RhIg given intravenously 53](#_Toc36199117)

[5.1.1. Results table 53](#_Toc36199118)

[5.1.2. GRADE 54](#_Toc36199119)

[5.1.3. Forest plot for Rh alloimmunisation at 6-9 months postpartum 56](#_Toc36199120)

# Postpartum administration

## 1.1 Postpartum RhIg compared to No postpartum treatment (or placebo)

### 1.1.1. Results table

| **Author Year, Country (study design)** | **Timepoint** | **RhIg dose** | **Postpartum RhIg** | **No RhIg/ placebo** | **Notes** |
| --- | --- | --- | --- | --- | --- |
| **Rh alloimmunisation after most recent pregnancy** | | | | | |
| Chown 1969 [1], Canada (RCT) | 6 months postpartum | 435 µg | 0/508^A^ | 36/500 |  |
| Combined study 1971 [2], UK and USA (RCT) | 6 months postpartum | 1000-5000 µg | 1/173  [1000 µg: 1/147;  1000-5000 µg ^B^: 0/26] | 38/176  [1000 µg: 30/145;  1000-5000 µg ^B^: 8/31] |  |
| Dudok De Wit 1968 [3], the Netherlands (RCT) | 4-6 months after birth | 250 µg/ml | 3/333 | 17/329 | Women and infants included irrespective of ABO compatibility. |
| White 1970 [4], International (RCT) | 6 months postpartum | any dose ^C^ | 6/3389 | 102/1476 | White 1970 was included in the ‘any dose’ meta-analysis (1.1.3), as doses varied. Ascari 1968 was included in the by dose meta-analysis (1.1.4). |
|  |  | 4000-6000 µg  (Ascari 1968 [5]) | 0/300 | 19/227 |  |
| Woodrow 1971 [6], UK (RCT) | 6 months postpartum | 200 µg | 0/353 | 13/362 |  |
| **Rh alloimmunisation at subsequent Rh-positive pregnancy** | | | | | |
| Combined study 1971 [2], UK and USA (RCT) | At end of the 2^nd^ Rh-positive pregnancy | 1000-5000 µg | 2(1)**^D^/**88  [1000 µg: 2/76;  1000-5000 µg ^B^: 0/12] | 20(12)^D^/65  [1000 µg: 19/61;  1000-5000 µg ^B^: 1/4] | . |
| White 1970 [4], International (RCT) | At delivery with subsequent Rh+ infant | any dose | 5/395 | 23(11)^E^/178 | White 1970 was included in the ‘any dose’ meta-analysis (1.1.5). |
| Woodrow 1971 [6], UK (RCT) | At end of 2^nd^ Rh-positive pregnancy | 200 µg | 3/128 | 13(4)^F^/127 |  |
| MacKenzie 1999 [7], UK (comparative cohort) | Second pregnancy | 500 IU | 26/3146 | 77/5971 | Women received postpartum dose only if the baby was Rh-positive. |

A Among those with concurrent controls.

B Given in Baltimore

C 306 women received 5000-7000 µg and 3083 received ≤300 µg

D Women who had developed antibodies after the first pregnancy are given in brackets

E 11 of 23 patients had detectable levels of anti-Rh 6 months after their first observed pregnancies.

F 4 women (brackets) who developed antibodies during the first pregnancy.

### 1.1.2. GRADE

#### Evidence profile

| **Postnatal RhIg compared to No postnatal RhIg (or placebo) to prevent Rh alloimmunisation** | | | | | | | | | | | |
| --- | --- | --- | --- | --- | --- | --- | --- | --- | --- | --- | --- |
| **Certainty assessment** | | | | | | | **№ of patients** | | **Effect** | | **Certainty** |
| **№ of studies [ref]** | **Study design** | **Risk of bias** | **Inconsistency** | **Indirectness** | **Imprecision** | **Other considerations** | **Postnatal RhIg** | **No RhIg (or placebo)** | **Relative (95% CI)** | **Absolute (95% CI)** |  |
| **Postnatal RhIg (any dose) vs No postnatal RhIg: up to 6 months follow-up** | | | | | | | | | | | |
| Rh alloimmunisation (ABO compatible) (follow up: 6 months) | | | | | | | | | | | |
| 4  [1,2,4,6] | randomised trials | very serious ^a^ | serious ^b^ | not serious | not serious ^c^ | none | 7/4423 (0.2%) | 189/2514 (7.5%) | **OR 0.07** (0.05 to 0.10) | **70 fewer per 1,000** (from 67 to 71 fewer) | ⨁◯◯◯ VERY LOW |
| Rh alloimmunisation (Irrespective of ABO status) (follow up: 6 months) | | | | | | | | | | | |
| 1  [3] | randomised trials | very serious ^d^ | not serious | serious ^e^ | serious ^f^ | none | 3/333 (0.9%) | 17/329 (5.2%) | **OR 0.23** (0.10 to 0.57) | **39 fewer per 1,000** (from 22 to 46 fewer) | ⨁◯◯◯ VERY LOW |
| **Postnatal RhIg (by dose) vs No postnatal RhIg: up to 6 months follow-up** | | | | | | | | | | | |
| Rh alloimmunisation [dose: 200 µg anti-D] (follow up: 6 months) | | | | | | | | | | | |
| 1  [6] | randomised trials | very serious ^d^ | not serious | not serious | serious ^f^ | none | 0/353 (0.0%) | 13/362 (3.6%) | **OR 0.13** (0.04 to 0.40) | **31 fewer per 1,000** (from 21 to 34 fewer) | ⨁◯◯◯ VERY LOW |
| Rh alloimmunisation [dose: ≤250 µg anti-D] (follow up: 6 months) | | | | | | | | | | | |
| 2  [3,6] | randomised trials | very serious ^d^ | not serious | not serious | serious ^f^ | none | 3/686 (0.4%) | 30/691 (4.3%) | **OR 0.19** (0.09 to 0.37) | **35 fewer per 1,000** (from 27 to 39 fewer) | ⨁◯◯◯ VERY LOW |
| Rh alloimmunisation [dose: 1000 µg of IgG anti-D] (follow up: 6 months) | | | | | | | | | | | |
| 1  [2] | randomised trials | very serious ^d^ | not serious | serious ^g^ | serious ^f^ | none | 1/147 (0.7%) | 30/145 (20.7%) | **OR 0.12** (0.06 to 0.26) | **177 fewer per 1,000** (from 143 to 191 fewer) | ⨁◯◯◯ VERY LOW |
| Rh alloimmunisation [dose: 1000 to 5000 µg of IgG anti-D] (follow up: 6 months) | | | | | | | | | | | |
| 1  [2] | randomised trials | very serious ^d^ | not serious | serious ^g^ | serious ^f^ | none | 0/26 (0.0%) | 8/31 (25.8%) | **OR 0.12** (0.03 to 0.54) | **218 fewer per 1,000** (from 100 to 248 fewer) | ⨁◯◯◯ VERY LOW |
| Rh alloimmunisation [dose: 4000-6000 µg anti-Rh] (follow up: 6 months) | | | | | | | | | | | |
| 1  [5] | randomised trials | serious ^h^ | not serious | serious ^g^ | serious ^f^ | none | 0/300 (0.0%) | 19/227 (8.4%) | **OR 0.09** (0.04 to 0.23) | **76 fewer per 1,000** (from 63 to 80 fewer) | ⨁◯◯◯ VERY LOW |
| **Postnatal RhIg (any dose) vs No postnatal RhIg: subsequent Rh-positive pregnancy** | | | | | | | | | | | |
| Rh alloimmunisation (follow up: subsequent Rh-positive pregnancy) | | | | | | | | | | | |
| 3  [2,4,6] | randomised trials | very serious ^a^ | not serious | not serious | serious ^f^ | none | 10/611 (1.6%) | 56/370 (15.1%) | **OR 0.12** (0.07 to 0.20) | **130 fewer per 1,000** (from 117 to 139 fewer) | ⨁◯◯◯ VERY LOW |
| 1  [7] | observational studies | extremely serious ^i^ | not serious | not serious | not serious | none | 26/3146 (0.8%) | 77/5971 (1.3%) | **OR 0.66** (0.44 to 0.99) | **4 fewer per 1,000** (from 0 to 7 fewer) | ⨁◯◯◯ VERY LOW |
| **Postnatal RhIg (by dose) vs No postnatal RhIg: subsequent Rh-positive pregnancy** | | | | | | | | | | | |
| Rh alloimmunisation [dose: 200 µg anti-D] (follow up: subsequent Rh-positive pregnancy) | | | | | | | | | | | |
| 1  [6] | randomised trials | very serious ^d^ | not serious | not serious | serious ^f^ | none | 3/128 (2.3%) | 13/127 (10.2%) | **OR 0.26** (0.10 to 0.72) | **74 fewer per 1,000** (from 26 to 91 fewer) | ⨁◯◯◯ VERY LOW |
| Rh alloimmunisation [dose: 1000 µg of IgG anti-D (UK)] (follow up: subsequent Rh-positive pregnancy) | | | | | | | | | | | |
| 1  [2] | randomised trials | very serious ^d^ | not serious | serious ^g^ | serious ^f^ | none | 2/76 (2.6%) | 19/61 (31.1%) | **OR 0.11** (0.04 to 0.29) | **264 fewer per 1,000** (from 195 to 294 fewer) | ⨁◯◯◯ VERY LOW |
| Rh alloimmunisation [dose: 1000 to 5000 µg of IgG anti-D (Baltimore)] (follow up: subsequent Rh-positive pregnancy) | | | | | | | | | | | |
| 1  [2] | randomised trials | very serious ^d^ | not serious | serious ^g^ | serious ^f^ | none | 0/12 (0.0%) | 1/4 (25.0%) | **OR 0.02** (0.00 to 1.69) | **243 fewer per 1,000** (from -- to 110 more) | ⨁◯◯◯ VERY LOW |

**CI:** Confidence interval; **OR:** Odds ratio

**Explanations**

a. Sequence generation was unclear or by quasi-randomised methods. Allocation concealment was not reported or not done due to quasi-randomisation methods.

b. High I^2^ values and statistically significant heterogeneity of effect estimates. This is due mainly from the White 1970 results which contribute almost half of the data.

c. Fewer events than the rule of thumb of 400 events, however, there is a sufficient number of participants (>2000). The relative CI does not cross the threshold of appreciable benefit, and the absolute CI is narrow.

d. Sequence generation based on non-randomised method and no allocation concealment.

e. ABO incompatibility between fetal erythrocytes and maternal serum partially protects the mother against Rh immunisation.

f. Few events (<rule of thumb of n=400 events) and small sample size (<2000 participants).

g. Dose larger than would be expected in the treatment group.

h. Insufficient information to evaluate most risk of bias domains.

i. No adjustment made for the detection and quantification of fetal-maternal hemorrhage, which was defined a priori as a variable that needed to be considered as part of an adjusted analysis.

#### Summary of Findings table

| **Outcomes** | **№ of participants (studies) Follow-up** | **Certainty of the evidence (GRADE)** | **Relative effect (95% CI)** | **Anticipated absolute effects** | |
| --- | --- | --- | --- | --- | --- |
|  |  |  |  | **Risk with No RhIg (or placebo)** | **Risk difference with Postpartum RhIg** |
| Rh alloimmunisation (ABO compatible) follow up: 6 months | 6937 (4 RCTs) | ⨁◯◯◯ VERY LOW ^a,b,c^ | **OR 0.07** (0.05 to 0.10) | 75 per 1,000 | **70 fewer per 1,000** (67 to 71 fewer) |
| Rh alloimmunisation (Irrespective of ABO status) follow up: 6 months | 662 (1 RCT) | ⨁◯◯◯ VERY LOW ^d,e,f^ | **OR 0.23** (0.10 to 0.57) | 52 per 1,000 | **39 fewer per 1,000** (22 to 46 fewer) |
| Rh alloimmunisation [dose: 200 µg anti-D] follow up: 6 months | 715 (1 RCT) | ⨁◯◯◯ VERY LOW ^d,f^ | **OR 0.13** (0.04 to 0.40) | 36 per 1,000 | **31 fewer per 1,000** (21 to 34 fewer) |
| Rh alloimmunisation [dose: ≤250 µg anti-D] follow up: 6 months | 1377 (2 RCTs) | ⨁◯◯◯ VERY LOW ^d,f^ | **OR 0.19** (0.09 to 0.37) | 43 per 1,000 | **35 fewer per 1,000** (27 to 39 fewer) |
| Rh alloimmunisation [dose: 1000 µg of IgG anti-D] follow up: 6 months | 292 (1 RCT) | ⨁◯◯◯ VERY LOW ^d,f,g^ | **OR 0.12** (0.06 to 0.26) | 207 per 1,000 | **177 fewer per 1,000** (143 to 191 fewer) |
| Rh alloimmunisation [dose: 1000 to 5000 µg of IgG anti-D] follow up: 6 months | 57 (1 RCT) | ⨁◯◯◯ VERY LOW ^d,f,g^ | **OR 0.12** (0.03 to 0.54) | 258 per 1,000 | **218 fewer per 1,000** (100 to 248 fewer) |
| Rh alloimmunisation [dose: 4000-6000 µg anti-Rh] follow up: 6 months | 527 (1 RCT) | ⨁◯◯◯ VERY LOW ^f,g,h^ | **OR 0.09** (0.04 to 0.23) | 84 per 1,000 | **76 fewer per 1,000** (63 to 80 fewer) |
| Rh alloimmunisation (follow up: subsequent Rh-positive pregnancy) | 981 (3 RCTs) | ⨁◯◯◯ VERY LOW ^a,f^ | **OR 0.12** (0.07 to 0.20) | 151 per 1,000 | **130 fewer per 1,000** (117 to 139 fewer) |
| Sensitisation in second pregnancy | 9117 (1 observational study) | ⨁◯◯◯ VERY LOW ^i^ | **OR 0.66** (0.44 to 0.99) | 13 per 1,000 | 4 fewer per 1,000 (0 to 7 fewer) |
| Rh alloimmunisation [dose: 200 µg anti-D] (follow up: subsequent Rh-positive pregnancy) | 255 (1 RCT) | ⨁◯◯◯ VERY LOW ^d,f^ | **OR 0.26** (0.10 to 0.72) | 102 per 1,000 | **74 fewer per 1,000** (26 to 91 fewer) |
| Rh alloimmunisation [dose: 1000 µg of IgG anti-D (UK)] (follow up: subsequent Rh-positive pregnancy) | 137 (1 RCT) | ⨁◯◯◯ VERY LOW ^d,f,g^ | **OR 0.11** (0.04 to 0.29) | 311 per 1,000 | **264 fewer per 1,000** (195 to 294 fewer) |
| Rh alloimmunisation [dose: 1000 to 5000 µg of IgG anti-D (Baltimore)] (follow up: subsequent Rh-positive pregnancy) | 16 (1 RCT) | ⨁◯◯◯ VERY LOW ^d,f,g^ | **OR 0.02** (0.00 to 1.69) | 250 per 1,000 | **243 fewer per 1,000** (250 fewer to 110 more) |
| ***The risk in the intervention group** (and its 95% confidence interval) is based on the assumed risk in the comparison group and the **relative effect** of the intervention (and its 95% CI).   **CI:** Confidence interval; **OR:** Odds ratio | | | | | |
| **GRADE Working Group grades of evidence** **High certainty:** We are very confident that the true effect lies close to that of the estimate of the effect **Moderate certainty:** We are moderately confident in the effect estimate: The true effect is likely to be close to the estimate of the effect, but there is a possibility that it is substantially different **Low certainty:** Our confidence in the effect estimate is limited: The true effect may be substantially different from the estimate of the effect **Very low certainty:** We have very little confidence in the effect estimate: The true effect is likely to be substantially different from the estimate of effect | | | | | |

**Explanations**

a. Sequence generation was unclear or by quasi-randomised methods. Allocation concealment was not reported or not done due to quasi-randomisation methods.

b. High I^2^ values and statistically significant heterogeneity of effect estimates. This is due mainly from the White 1970 results which contribute almost half of the data.

c. Fewer events than the rule of thumb of 400 events, however, there is a sufficient number of participants (>2000). The relative CI does not cross the threshold of appreciable benefit, and the absolute CI is narrow.

d. Sequence generation based on non-randomised method and no allocation concealment.

e. ABO incompatibility between fetal erythrocytes and maternal serum partially protects the mother against Rh immunisation.

f. Few events (<rule of thumb of n=400 events) and small sample size (<2000 participants).

g. Dose larger than would be expected in the treatment group.

h. Insufficient information to evaluate most risk of bias domains.

i. No adjustment made for the detection and quantification of fetal-maternal hemorrhage, which was defined a priori as a variable that needed to be considered as part of an adjusted analysis.

### 1.1.3. Forest plot for Rh alloimmunisation at 6 months postpartum (any dose)

*Comparison: Postpartum RhIg (any dose) vs No RhIg*


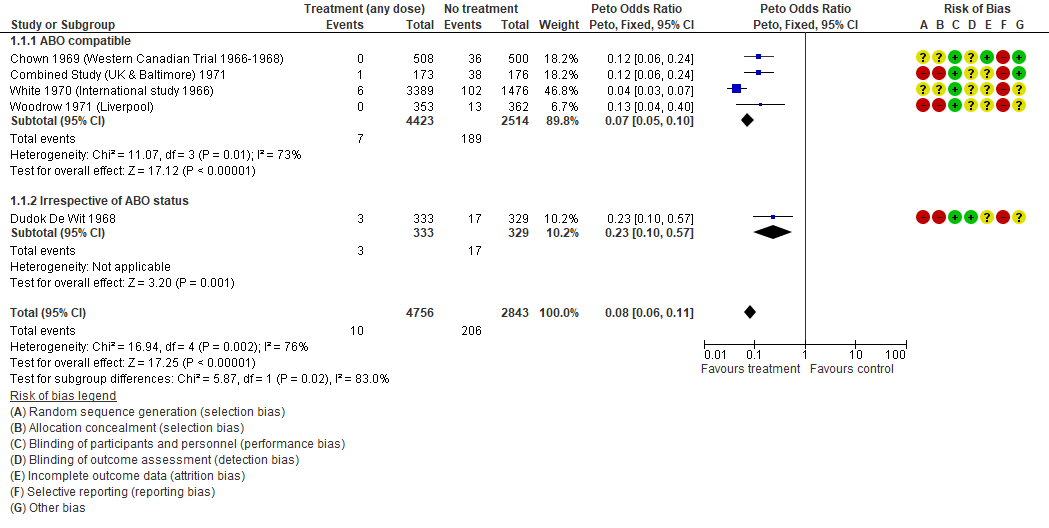


**Risk of bias legend**: (A) Random sequence generation (selection bias); (B) Allocation concealment (selection bias); (C) Blinding of participants and personnel (performance bias); (D) Blinding of outcome assessment (detection bias); (E) Incomplete outcome data (attrition bias); (F) Selective reporting (reporting bias); (G) Other bias

### 1.1.4. Forest plot for Rh alloimmunisation at 6 months postpartum (by dose)

*Comparison: Postpartum RhIg (by dose) vs No postpartum RhIg*


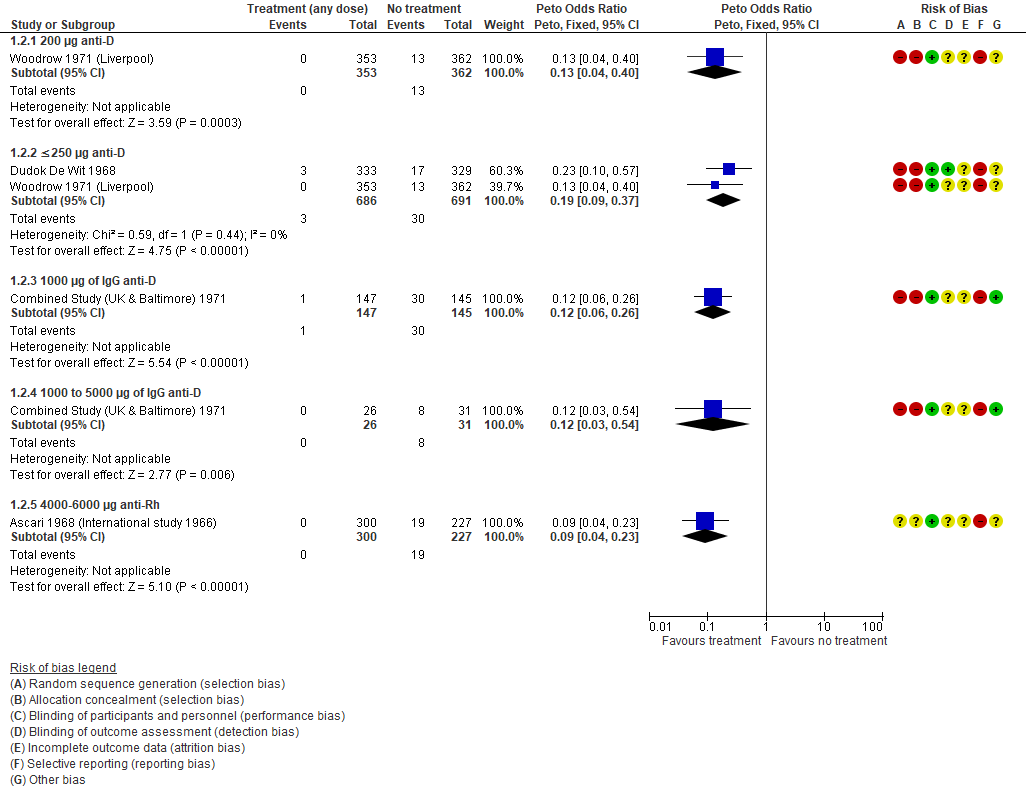


**Risk of bias legend**: (A) Random sequence generation (selection bias); (B) Allocation concealment (selection bias); (C) Blinding of participants and personnel (performance bias); (D) Blinding of outcome assessment (detection bias); (E) Incomplete outcome data (attrition bias); (F) Selective reporting (reporting bias); (G) Other bias

### 1.1.5. Forest plot for Rh alloimmunisation at subsequent Rh-positive pregnancy (any dose)

*Comparison: Postpartum RhIg (any dose) vs No postpartum RhIg*


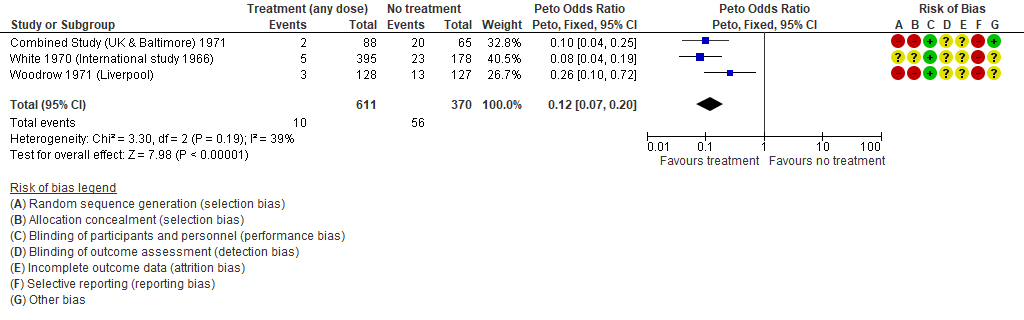


**Risk of bias legend**: (A) Random sequence generation (selection bias); (B) Allocation concealment (selection bias); (C) Blinding of participants and personnel (performance bias); (D) Blinding of outcome assessment (detection bias); (E) Incomplete outcome data (attrition bias); (F) Selective reporting (reporting bias); (G) Other bias

### 1.1.6. Forest plot for Rh alloimmunisation at subsequent Rh-positive pregnancy (by dose)

*Comparison: Postpartum RhIg (by dose) vs No postpartum RhIg*


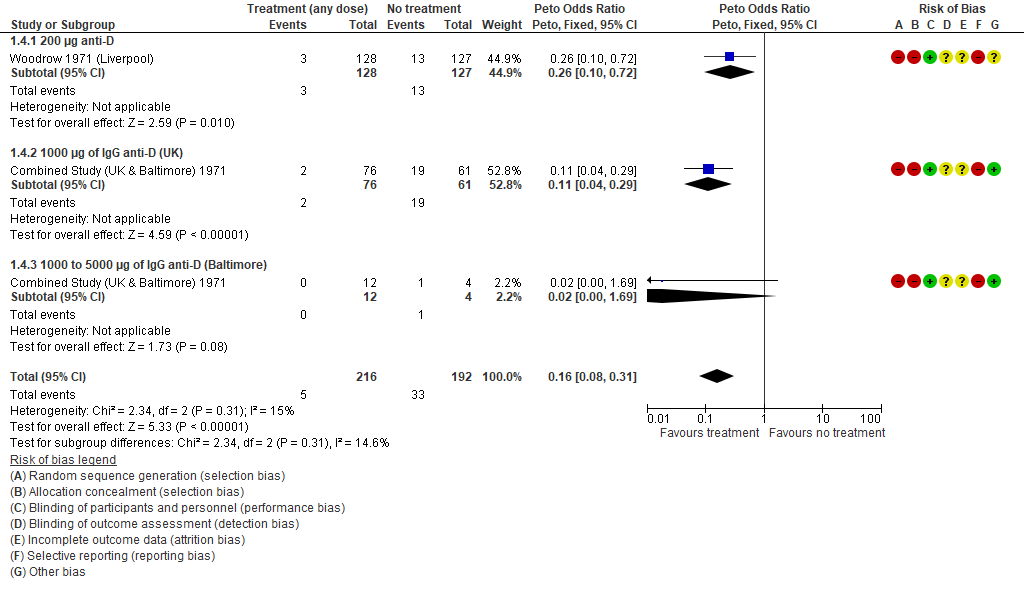


**Risk of bias legend**: (A) Random sequence generation (selection bias); (B) Allocation concealment (selection bias); (C) Blinding of participants and personnel (performance bias); (D) Blinding of outcome assessment (detection bias); (E) Incomplete outcome data (attrition bias); (F) Selective reporting (reporting bias); (G) Other bias

### 1.1.7. Forest plot for sensitisation in second pregnancy (observational data)

*Comparison: Postnatal RhIg vs No RhIg*


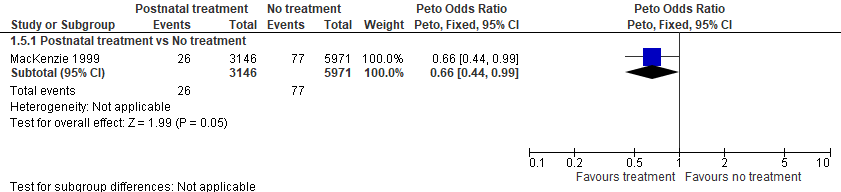


## Higher-dose postnatal RhIg compared to Lower-dose postnatal RhIg

### 1.2.1. Results table

| **Author Year, Country (study design)** | **Timepoint** | **Comparison** | **Higher-dose RhIg** | **Lower-dose RhIg** | **Notes** |  |
| --- | --- | --- | --- | --- | --- | --- |
| **Rh alloimmunisation after most recent pregnancy** | | | | | | |
| Medical Research Council 1974 [8] ^A^, UK (RCT) | 6 months after delivery of first infant | ≥50 µg vs <50 µg | 4/1354 | 6/446 | Higher dose RhIg ranged from 50-200ug/2ml. Lower dose was 20 µg. |  |
| Medical Research Council 1974 [8] ^A^, UK (RCT) | 6 months after delivery of first infant | ≥100 µg vs <100 µg | 2/902 | 8/898 | Higher dose RhIg ranged from 100-200ug/2ml. |  |
| Medical Research Council 1974 [8] ^A^, UK (RCT) | 6 months after delivery of first infant | ≥200 µg vs <200 µg | 1/459 | 9/1341 | Higher dose RhIg was 200ug/2ml. |  |
| Ascari 1968 [5], International (RCT) | 6 months | ≥4000 µg vs <4000 µg | 0/300 | 1/781 | Higher dose was 4000-6000 µg total (1000ug to 1200ug of anti-Rh/ml in a 5 ml injection. |  |
| **Rh alloimmunisation at the end of a second d-positive pregnancy** | | | | | | |
| Medical Research Council 1974 [8] ^A^, UK (RCT) | End of second d-positive pregnancy | ≥50 µg vs <50 µg | 8(1)^B^/656 | 6(5)^B^/205 | Higher dose RhIg ranged from 50-200ug/2ml. Lower dose was 20 µg. |  |
| Medical Research Council 1974 [8] ^A^, UK (RCT) | End of second d-positive pregnancy | ≥100 µg vs <100 µg | 5(1)^B^/395 | 9(5)^B^/412 | Higher dose RhIg ranged from 100-200ug/2ml. |  |
| Medical Research Council 1974 [8] ^A^, UK (RCT) | End of second d-positive pregnancy | ≥200 µg vs <200 µg | 3(1)/206 | 11(5)^B^/601 | Higher dose RhIg was 200ug/2ml. |  |

A Study also reports the results among those who were positive only with enzyme treated red cells, usually only with undiluted serum [results not reported here]. Anti-D detected using IAGT.

B Women who were positive at 6 months after the first pregnancy. Anti-D detected using IAGT.

### 1.2.2. GRADE

#### Evidence profile

| **Higher-dose postnatal RhIg compared to Lower-dose postnatal RhIg for prevention of Rh alloimmunisation** | | | | | | | | | | | |
| --- | --- | --- | --- | --- | --- | --- | --- | --- | --- | --- | --- |
| **Certainty assessment** | | | | | | | **№ of patients** | | **Effect** | | **Certainty** |
| **№ of studies**  **[ref]** | **Study design** | **Risk of bias** | **Inconsistency** | **Indirectness** | **Imprecision** | **Other considerations** | **Higher-dose postnatal RhIg** | **Lower-dose postnatal RhIg** | **Relative (95% CI)** | **Absolute (95% CI)** |  |
| **Higher-dose postnatal RhIg vs Lower-dose postnatal RhIg: 6 months follow-up** | | | | | | | | | | | |
| Rh alloimmunisation [dose: 50 µg vs <50 µg] (follow up: 6 months) | | | | | | | | | | | |
| 1  [8] | randomised trials | serious ^a^ | not serious | serious ^b^ | serious ^c^ | none | 2/452 (0.4%) | 6/446 (1.3%) | **OR 0.36** (0.09 to 1.45) | **9 fewer per 1,000** (from 12 fewer to 6 more) | ⨁◯◯◯ VERY LOW |
| Rh alloimmunisation [dose: 100 µg vs <100 µg] (follow up: 6 months) | | | | | | | | | | | |
| 1  [8] | randomised trials | serious ^a^ | not serious | serious ^b^ | serious ^c^ | none | 1/443 (0.2%) | 8/898 (0.9%) | **OR 0.37** (0.09 to 1.49) | **6 fewer per 1,000** (8 fewer to 4 more) | ⨁◯◯◯ VERY LOW |
| Rh alloimmunisation [dose: 200 µg vs <200 µg] (follow up: 6 months) | | | | | | | | | | | |
| 1  [8] | randomised trials | serious ^a^ | not serious | not serious | serious ^c^ | none | 1/459 (0.2%) | 9/1341 (0.7%) | **OR 0.44** (0.11 to 1.83) | **4 fewer per 1,000** (from 6 fewer to 6 more) | ⨁⨁◯◯ LOW |
| Rh alloimmunisation [dose: 4000-6000 µg vs <4000 µg] (follow up: 6 months) | | | | | | | | | | | |
| 1  [5] | randomised trials | serious ^d^ | not serious | serious ^e^ | serious ^c^ | none | 0/300 (0.0%) | 1/781 (0.1%) | **OR 0.25** (0.00 to 19.95) | **1 fewer per 1,000** (from -- to 24 more) | ⨁◯◯◯ VERY LOW |
| **Higher-dose postnatal RhIg vs Lower-dose postnatal RhIg: end of second d-positive pregnancy** | | | | | | | | | | | |
| Rh alloimmunisation [dose: 50 µg vs <50 µg] (follow up: end of second d-positive pregnancy) | | | | | | | | | | | |
| 1  [8] | randomised trials | serious ^a^ | not serious | serious ^b^ | serious ^c^ | none | 3/207 (1.4%) | 6/205 (2.9%) | **OR 0.50** (0.13 to 1.88) | **14 fewer per 1,000** (from 25 fewer to 24 more) | ⨁◯◯◯ VERY LOW |
| Rh alloimmunisation [dose: 100 µg vs <100 µg] (follow up: end of second d-positive pregnancy) | | | | | | | | | | | |
| 1  [8] | randomised trials | serious ^a^ | not serious | serious ^b^ | serious ^c^ | none | 2/189 (1.1%) | 9/412 (2.2%) | **OR 0.53** (0.15 to 1.93) | **10 fewer per 1,000** (from 19 fewer to 19 more) | ⨁◯◯◯ VERY LOW |
| Rh alloimmunisation [dose: 200 µg vs <200 µg] (follow up: end of second d-positive pregnancy) | | | | | | | | | | | |
| 1  [8] | randomised trials | serious ^a^ | not serious | not serious | serious ^c^ | none | 3/206 (1.5%) | 11/601 (1.8%) | **OR 0.80** (0.24 to 2.70) | **4 fewer per 1,000** (from 14 fewer to 30 more) | ⨁⨁◯◯ LOW |

**CI:** Confidence interval; **OR:** Odds ratio

**Explanations**

a. Allocation concealment and blinding of outcome assessment not reported.

b. Dose lower than would be expected in lower treatment group.

c. Few events (<rule of thumb of n=400 events) and small sample size (<2000 participants).

d. Insufficient information reported in multiple domains to provide a judgement.

e. Dose larger than would be expected in treatment group

#### Summary of Findings table

| **Outcomes** | **№ of participants (studies) Follow-up** | **Certainty of the evidence (GRADE)** | **Relative effect (95% CI)** | **Anticipated absolute effects** | |
| --- | --- | --- | --- | --- | --- |
|  |  |  |  | **Risk with lower RhIg doses given at delivery** | **Risk difference with Higher RhIg** |
| Rh alloimmunisation [dose: ≥50 µg vs <50 µg] follow up: 6 months | 1800 (1 RCT) | ⨁◯◯◯ VERY LOW ^a,b,c^ | **OR 0.15** (0.04 to 0.63) | 13 per 1,000 | **11 fewer per 1,000** (5 to 13 fewer) |
| Rh alloimmunisation [dose: ≥100 µg vs <100 µg] follow up: 6 months | 1800 (1 RCT) | ⨁◯◯◯ VERY LOW ^a,b,c^ | **OR 0.30** (0.09 to 1.03) | 9 per 1,000 | **6 fewer per 1,000** (0 to 8 fewer) |
| Rh alloimmunisation [dose: ≥200 µg vs <200 µg] follow up: 6 months | 1800 (1 RCT) | ⨁⨁◯◯ LOW ^a,c^ | **OR 0.44** (0.11 to 1.83) | 7 per 1,000 | **4 fewer per 1,000** (6 fewer to 6 more) |
| Rh alloimmunisation [dose: ≥4000 µg vs <4000 µg] follow up: 6 months | 1081 (1 RCT) | ⨁◯◯◯ VERY LOW ^c,d,e^ | **OR 0.25** (0.00 to 19.95) | 1 per 1,000 | **1 fewer per 1,000** (1 fewer to 24 more) |
| Rh alloimmunisation [dose: ≥50 µg vs <50 µg] (follow up: end of second d-positive pregnancy) | 861 (1 RCT) | ⨁◯◯◯ VERY LOW ^a,b,c^ | **OR 0.34** (0.10 to 1.19) | 29 per 1,000 | **19 fewer per 1,000** (26 fewer to 5 more) |
| Rh alloimmunisation [dose: ≥100 µg vs <100 µg] (follow up: end of second d-positive pregnancy) | 807 (1 RCT) | ⨁◯◯◯ VERY LOW ^a,b,c^ | **OR 0.58** (0.20 to 1.68) | 22 per 1,000 | **9 fewer per 1,000** (17 fewer to 14 more) |
| Rh alloimmunisation [dose: ≥200 µg vs <200 µg] (follow up: end of second d-positive pregnancy) | 807 (1 RCT) | ⨁⨁◯◯ LOW ^a,c^ | **OR 0.80** (0.24 to 2.70) | 18 per 1,000 | **4 fewer per 1,000** (14 fewer to 30 more) |
| ***The risk in the intervention group** (and its 95% confidence interval) is based on the assumed risk in the comparison group and the **relative effect** of the intervention (and its 95% CI).   **CI:** Confidence interval; **OR:** Odds ratio | | | | | |
| **GRADE Working Group grades of evidence** **High certainty:** We are very confident that the true effect lies close to that of the estimate of the effect **Moderate certainty:** We are moderately confident in the effect estimate: The true effect is likely to be close to the estimate of the effect, but there is a possibility that it is substantially different **Low certainty:** Our confidence in the effect estimate is limited: The true effect may be substantially different from the estimate of the effect **Very low certainty:** We have very little confidence in the effect estimate: The true effect is likely to be substantially different from the estimate of effect | | | | | |

**Explanations**

a. Allocation concealment and blinding of outcome assessment not reported.

b. Dose lower than would be expected in lower treatment group.

c. Few events (<rule of thumb of n=400 events) and small sample size (<2000 participants).

d. Insufficient information reported in multiple domains to provide a judgement.

e. Dose larger than would be expected in treatment group.

### 1.2.3. Forest plot for Rh alloimmunisation at 6 months postpartum

*Comparison: Higher-dose postnatal RhIg vs Lower-dose postnatal RhIg*


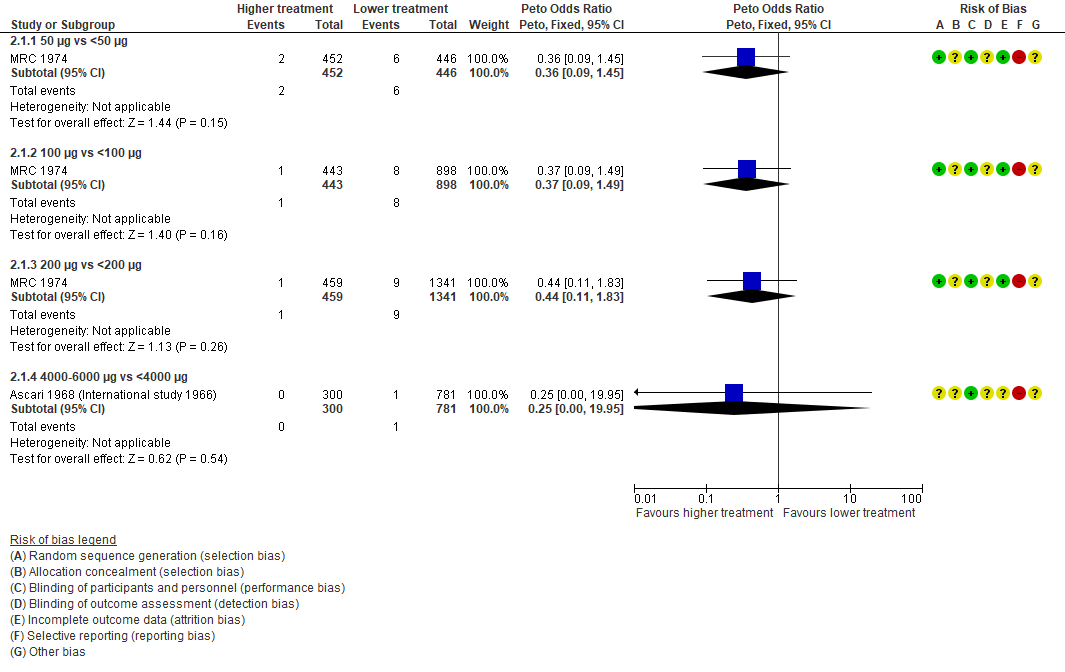


**Risk of bias legend**: (A) Random sequence generation (selection bias); (B) Allocation concealment (selection bias); (C) Blinding of participants and personnel (performance bias); (D) Blinding of outcome assessment (detection bias); (E) Incomplete outcome data (attrition bias); (F) Selective reporting (reporting bias); (G) Other bias

### 1.2.4. Forest plot for Rh alloimmunisation at end of second d-positive pregnancy

*Comparison: Higher-dose postnatal RhIg vs Lower-dose postnatal RhIg*


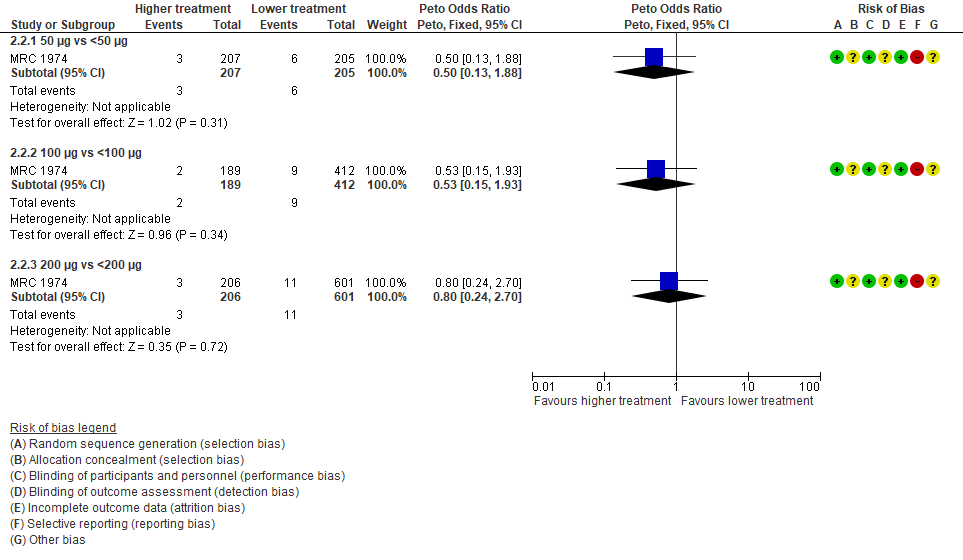


**Risk of bias legend**: (A) Random sequence generation (selection bias); (B) Allocation concealment (selection bias); (C) Blinding of participants and personnel (performance bias); (D) Blinding of outcome assessment (detection bias); (E) Incomplete outcome data (attrition bias); (F) Selective reporting (reporting bias); (G) Other bias

# 2. Antenatal administration

## 2.1 Antenatal RhIg (any dose) vs No antenatal RhIg

### 2.1.1. Results table

| **Author Year, Country (study design)** | **Timepoint** | **Antenatal RhIg**  **(28 and 34 weeks)** | **No RhIg** | **Notes** |  |
| --- | --- | --- | --- | --- | --- |
| **Rh alloimmunisation at delivery** | | | | | |
| Huchet 1987 [9], France (RCT) | at delivery | 1/599^A^  [1/927]^B^ | 6/590^A^  [6/955]^B^ | 100 µg at each administration |  |
| Lee 1995 [10], UK (RCT) | at delivery | 4/513^A^  [4/952]^B^ | 7/595^A^  [7/1068]^B^ | 50 µg at each administration |  |
| **Rh alloimmunisation 2-12 months postpartum** | | | | | |
| Huchet 1987 [9], France (RCT) | 2-12 months postpartum | 1/472^A^ | 7/468^A^ | 100 µg at each administration |  |
| Lee 1995 [10], UK (RCT) | 6 months postpartum | 5/513^A^  [5/952]^B^ | 9/595^A^  [9/1068]^B^ | 50 µg at each administration |  |
| **Rh alloimmunisation at delivery and after delivery** | | | | | |
| Huchet 1987 [9], France (RCT) | at delivery and at 2 to 12 months follow-up | 1/599^A^  [1/927]^B^ | 7/590^A^  [7/955]^B^ | 100 µg at each administration |  |

A among women with Rh-D positive infants

B among all treated women (i.e., regardless of Rh status of infant)

### 2.1.2. GRADE

#### Evidence profile

| **Antenatal RhIg (any dose) compared to No antenatal RhIg to prevent Rh alloimmunisation** | | | | | | | | | | | | | | |
| --- | --- | --- | --- | --- | --- | --- | --- | --- | --- | --- | --- | --- | --- | --- |
| **Certainty assessment** | | | | | | | **№ of patients** | | | **Effect** | | | **Certainty** |  |
| **№ of studies**  **[ref]** | **Study design** | **Risk of bias** | **Inconsistency** | **Indirectness** | **Imprecision** | **Other considerations** | | **Antenatal RhIg** | **No RhIg** | | **Relative (95% CI)** | **Absolute (95% CI)** |  |  |
| **Antenatal RhIg vs No antenatal RhIg: at delivery** | | | | | | | | | | | | | |  |
| Rh alloimmunisation [Among all treated women] (at delivery) | | | | | | | | | | | | | |  |
| 2  [9,10] | randomised trials | very serious ^a,b,c^ | not serious | serious ^d^ | serious ^e^ | none | | 5/1879 (0.3%) | 13/2023 (0.6%) | | **OR 0.44** (0.18 to 1.12) | **4 fewer per 1,000** (from 5 fewer to 1 more) | ⨁◯◯◯ VERY LOW |  |
| Rh alloimmunisation [Among those with Rh-D positive infant] (at delivery) | | | | | | | | | | | | | |  |
| 2  [9,10] | randomised trials | very serious ^a,b,c^ | not serious | not serious | serious ^e^ | none | | 5/1112 (0.4%) | 13/1185 (1.1%) | | **OR 0.44** (0.18 to 1.12) | **6 fewer per 1,000** (from 9 fewer to 1 more) | ⨁◯◯◯ VERY LOW |  |
| **Antenatal RhIg vs No antenatal RhIg: 2 to 12 months postpartum** | | | | | | | | | | | | | |  |
| Rh alloimmunisation [Among all treated women] (follow up: up to 12 months postpartum) | | | | | | | | | | | | | |  |
| 1  [9] | randomised trials | very serious ^b,c^ | not serious | serious ^d^ | serious ^e^ | none | | 5/952 (0.5%) | 9/1068 (0.8%) | | **OR 0.63** (0.22 to 1.81) | **3 fewer per 1,000** (from 7 fewer to 7 more) | ⨁◯◯◯ VERY LOW |  |
| Rh alloimmunisation [Among those with Rh-positive infant] (follow up: up to 12 months postpartum) | | | | | | | | | | | | | |  |
| 2  [9,10] | randomised trials | very serious ^a,b,c^ | not serious | not serious | serious ^e^ | none | | 6/985 (0.6%) | 16/1063 (1.5%) | | **OR 0.44** (0.19 to 1.01) | **8 fewer per 1,000** (from 0 to 12 fewer) | ⨁◯◯◯ VERY LOW |  |
| **Antenatal RhIg vs No antenatal RhIg: at delivery and at 2 to 12 months follow-up** | | | | | | | | | | | | | |  |
| Rh alloimmunisation [Among all treated women] (at delivery and at 2 to 12 months follow-up) | | | | | | | | | | | | | |  |
| 1  [9] | randomised trials | very serious ^a,c^ | not serious | serious ^d^ | serious ^f^ | none | | 1/927 (0.1%) | 7/955 (0.7%) | | **OR 0.23**  (0.06 to 0.92) | **6 fewer per 1,000**  (from 1 to 7 fewer) | ⨁◯◯◯ VERY LOW |  |
| Rh alloimmunisation [Among those with Rh-positive infant] (at delivery and at 2 to 12 months follow-up) | | | | | | | | | | | | | |  |
| 1  [9] | randomised trials | very serious ^a,c^ | not serious | not serious | serious ^f^ | none | | 1/599 (0.2%) | 7/590 (1.2%) | | **OR 0.22**  (0.05 to 0.87) | **9 fewer per 1,000**  (from 2 to 11 fewer) | ⨁◯◯◯ VERY LOW |  |

**CI:** Confidence interval; **OR:** Odds ratio

**Explanations**

a. Sequence generation not proper randomisation method and no allocation concealment.

b. Insufficient information provided to make a judgement on sequence generation and allocation concealment.

c. Many women lost to follow up with no reasons provided.

d. Includes women who delivered Rh-negative babies.

e. The number of events is less than the rule of thumb of 400 events, but the number of participants is large (>2000 participants). However, both the relative and absolute CIs include no effect.

f. The number of events is less than the rule of thumb of 400 events and the number of participants is <2000.

#### Summary of Findings table

| **Outcomes** | **№ of participants (studies) Follow-up** | **Certainty of the evidence (GRADE)** | **Relative effect (95% CI)** | **Anticipated absolute effects** | |
| --- | --- | --- | --- | --- | --- |
|  |  |  |  | **Risk with no antenatal RhIg** | **Risk difference with antenatal RhIg** |
| Rh alloimmunisation [Among all treated women] (at delivery) | 3902 (2 RCTs) | ⨁◯◯◯ VERY LOW ^a,b,c,d,e^ | **OR 0.44** (0.18 to 1.12) | 6 per 1,000 | **4 fewer per 1,000** (5 fewer to 1 more) |
| Rh alloimmunisation [Among those with Rh-D positive infants] (at delivery) | 2297 (2 RCTs) | ⨁◯◯◯ VERY LOW ^a,b,c,e^ | **OR 0.44** (0.18 to 1.12) | 11 per 1,000 | **6 fewer per 1,000** (9 fewer to 1 more) |
| Rh alloimmunisation [Among all treated] (follow up: up to 12 months postpartum) | 2020 (1 RCT) | ⨁◯◯◯ VERY LOW ^b,c,d,e^ | **OR 0.63** (0.22 to 1.81) | 8 per 1,000 | **3 fewer per 1,000** (7 fewer to 7 more) |
| Rh alloimmunisation [Among those with Rh-positive infant] (follow up: up to 12 months postpartum) | 2048 (2 RCTs) | ⨁◯◯◯ VERY LOW ^a,b,c,e^ | **OR 0.44** (0.19 to 1.01) | 15 per 1,000 | **8 fewer per 1,000** (0 to 12 fewer) |
| Rh alloimmunisation [Among all treated women] (at delivery and at 2-12 months follow-up) | 1882 (1 RCT) | ⨁◯◯◯ VERY LOW ^a,c,d,f^ | **OR 0.23** (0.06 to 0.92) | 7 per 1,000 | **6 fewer per 1,000** (1 to 7 fewer) |
| Rh alloimmunisation [Among those with Rh-positive infant who were test at delivery] (at delivery and at 2-12 months follow-up) | 1189 (1 RCT) | ⨁◯◯◯ VERY LOW ^a,c,d,f^ | **OR 0.22** (0.05 to 0.87) | 12 per 1,000 | **9 fewer per 1,000** (2 to 11 fewer) |
| ***The risk in the intervention group** (and its 95% confidence interval) is based on the assumed risk in the comparison group and the **relative effect** of the intervention (and its 95% CI).   **CI:** Confidence interval; **OR:** Odds ratio | | | | | |
| **GRADE Working Group grades of evidence** **High certainty:** We are very confident that the true effect lies close to that of the estimate of the effect **Moderate certainty:** We are moderately confident in the effect estimate: The true effect is likely to be close to the estimate of the effect, but there is a possibility that it is substantially different **Low certainty:** Our confidence in the effect estimate is limited: The true effect may be substantially different from the estimate of the effect **Very low certainty:** We have very little confidence in the effect estimate: The true effect is likely to be substantially different from the estimate of effect | | | | | |

**Explanations**

a. Sequence generation not proper randomisation method and no allocation concealment.

b. Insufficient information provided to make a judgement on sequence generation and allocation concealment.

c. Many women lost to follow up with no reasons provided.

d. Includes women who delivered Rh-negative babies.

e. The number of events is less than the rule of thumb of 400 events, but the number of participants is large (>2000). However, both the relative and absolute CIs include no effect.

f. The number of events is less than the rule of thumb of 400 events and the number of participants is <2000.

### 2.1.3. Forest plot for Rh alloimmunisation (among those with Rh-positive infants)


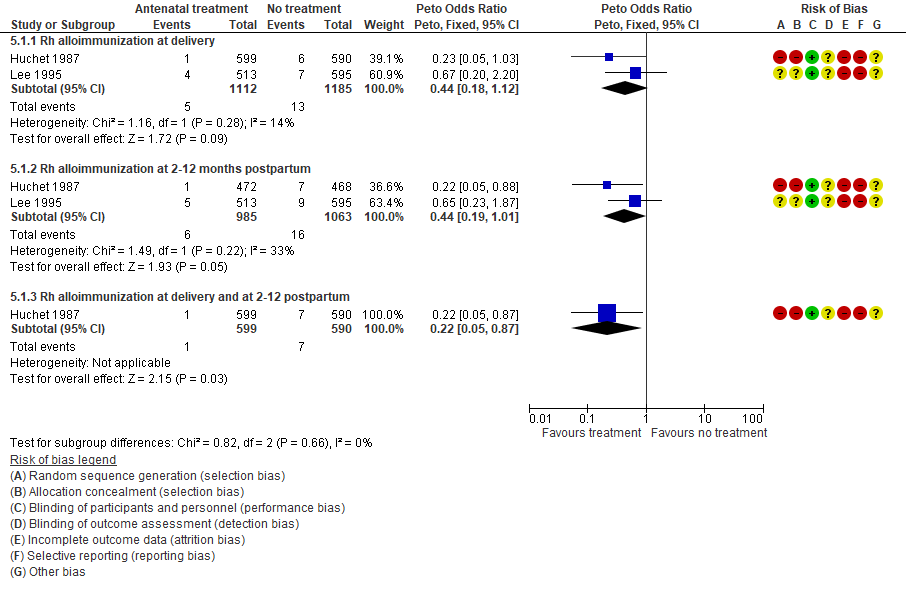


**Risk of bias legend**: (A) Random sequence generation (selection bias); (B) Allocation concealment (selection bias); (C) Blinding of participants and personnel (performance bias); (D) Blinding of outcome assessment (detection bias); (E) Incomplete outcome data (attrition bias); (F) Selective reporting (reporting bias); (G) Other bias

## 2.2 Antenatal plus postnatal RhIg vs No RhIg

### 2.2.1. Results table

| **Author Year, Country (study design)** | **Timepoint** | **Antenatal plus postnatal RhIg** | **No RhIg** | **Notes** |  |
| --- | --- | --- | --- | --- | --- |
| **Sensitisation in second pregnancy** | | | | | |
| MacKenzie 1999 [7], UK (comparative cohort) | Second pregnancy | 12/3320 | 77/5971 | Women received the postnatal dose of 500 IU regardless of the baby’s Rh status. |  |

### 2.2.2 GRADE

#### Evidence profile

| **Certainty assessment** | | | | | | | **№ of patients** | | **Effect** | | **Certainty** |
| --- | --- | --- | --- | --- | --- | --- | --- | --- | --- | --- | --- |
| **№ of studies**  **[ref]** | **Study design** | **Risk of bias** | **Inconsistency** | **Indirectness** | **Imprecision** | **Other considerations** | **Antenatal plus postnatal RhIg** | **No RhIg** | **Relative (95% CI)** | **Absolute (95% CI)** |  |
| Sensitisation in second pregnancy | | | | | | | | | | | |
| 1  [7] | observational studies | extremely serious ^a^ | not serious | serious ^b^ | not serious | none | 12/3320 (0.4%) | 77/5971 (1.3%) | **OR 0.38** (0.24 to 0.58) | **8 fewer per 1,000** (from 5 to 10 fewer) | ⨁◯◯◯ VERY LOW |

**CI:** Confidence interval; **OR:** Odds ratio

**Explanations**

a. No adjustment made for the detection and quantification of fetal-maternal hemorrhage, which was defined a priori as a variable that needed to be considered as part of an adjusted analysis.

b. Those in the antenatal plus postnatal treatment group were given postnatal prophylaxis regardless of the baby's Rh status.

#### Summary of findings

| **Outcomes** | **№ of participants (studies) Follow-up** | **Certainty of the evidence (GRADE)** | **Relative effect (95% CI)** | **Anticipated absolute effects** | |
| --- | --- | --- | --- | --- | --- |
|  |  |  |  | **Risk with no RhIg** | **Risk difference with antenatal plus postnatal RhIg** |
| Sensitisation in second pregnancy (after antenatal and postnatal RhIg) | 9291 (1 observational study) | ⨁◯◯◯ VERY LOW ^a,b^ | **OR 0.38** (0.24 to 0.58) | 13 per 1,000 | 8 fewer per 1,000 (5 to 10 fewer) |
| ***The risk in the intervention group** (and its 95% confidence interval) is based on the assumed risk in the comparison group and the **relative effect** of the intervention (and its 95% CI).   **CI:** Confidence interval; **OR:** Odds ratio | | | | | |
| **GRADE Working Group grades of evidence** **High certainty:** We are very confident that the true effect lies close to that of the estimate of the effect **Moderate certainty:** We are moderately confident in the effect estimate: The true effect is likely to be close to the estimate of the effect, but there is a possibility that it is substantially different **Low certainty:** Our confidence in the effect estimate is limited: The true effect may be substantially different from the estimate of the effect **Very low certainty:** We have very little confidence in the effect estimate: The true effect is likely to be substantially different from the estimate of effect | | | | | |

**Explanations**

a. No adjustment made for the detection and quantification of fetal-maternal hemorrhage, which was defined a priori as a variable that needed to be considered as part of an adjusted analysis.

b. Those in the antenatal plus postnatal treatment group were given postnatal prophylaxis regardless of the baby's Rh status.

### 2.2.3. Forest plot for sensitisation in second pregnancy

*Comparison: Antenatal plus postnatal RhIg vs No RhIg*


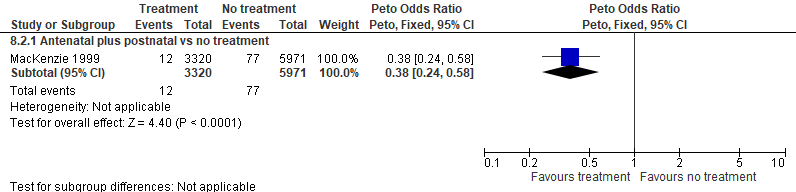


## 2.3 Two-dose antenatal RhIg vs One-dose antenatal RhIg

### 2.3.1. Results table

| **Author Year, Country (study design)** | **Timepoint** | **Two-dose regimen**  **(28 and 34 weeks)** | **One-dose regiment**  **(28 weeks; 1500 IU)** | **Notes** |  |
| --- | --- | --- | --- | --- | --- |
| **Major adverse events** | | | | | |
| White 2019 [11], Australia (RCT) | Not reported | 0/139 (625 IU/dose) | 0/138 | Authors noted that "The greater injection volume (>5 mL) for the single dose group initially made it more painful than for the standard regimen; the problem was alleviated by using a more concentrated product, delivering the same dose in a smaller volume (2 mL)." |  |

## 2.4 Two-dose antenatal plus postnatal RhIg vs One-dose antenatal plus postnatal RhIg

### 2.4.1. Results table

| **Author Year** | **Timepoint** | **Two-dose antenatal RhIg (28 & 34 wks)** | **One-dose antenatal RhIg (28 or 34 wks)** | **Notes** |  |
| --- | --- | --- | --- | --- | --- |
| **Rh alloimmunisation** | | | | | |
| Bowman 1978 [12], Canada (comparative cohort) | At delivery | 0/1204 | 0/153 | Dose of 300 µg. All women were given postnatal Rh immune globulin if they delivered a Rh-positive baby. |  |
|  | 6 months postpartum | 0/1204 | 0/153 |  |  |

### 2.4.2. GRADE

#### Evidence profile

| **Certainty assessment** | | | | | | | **№ of patients** | | **Effect** | | **Certainty** |
| --- | --- | --- | --- | --- | --- | --- | --- | --- | --- | --- | --- |
| **№ of studies**  **[ref]** | **Study design** | **Risk of bias** | **Inconsistency** | **Indirectness** | **Imprecision** | **Other considerations** | **Two-dose antenatal RhIg** | **One-dose antenatal RhIg** | **Relative (95% CI)** | **Absolute (95% CI)** |  |
| Rh alloimmunisation at delivery | | | | | | | | | | | |
| 1  [12] | observational studies | extremely serious ^a^ | not serious | not serious | not serious ^b^ | none | 0/1204 (0.0%) | 0/153 (0.0%) | not estimable | - | ⨁◯◯◯ VERY LOW |
| Rh alloimmunisation (follow up: 6 months) | | | | | | | | | | | |
| 1  [12] | observational studies | extremely serious ^a^ | not serious | not serious | not serious ^b^ | none | 0/1204 (0.0%) | 0/153 (0.0%) | not estimable | - | ⨁◯◯◯ VERY LOW |

**CI:** Confidence interval

**Explanations**

a. No adjustment made for the detection and quantification of fetal-maternal hemorrhage, which was defined a priori as a variable that needed to be considered as part of an adjusted analysis.

b. No events in either group, so not evaluated.

#### Summary of findings table

| **Outcomes** | **№ of participants (studies) Follow-up** | **Certainty of the evidence (GRADE)** | **Relative effect (95% CI)** | **Anticipated absolute effects** | |
| --- | --- | --- | --- | --- | --- |
|  |  |  |  | **Risk with one-dose antenatal (plus postnatal) RhIg** | **Risk difference with two-dose antenatal (plus postnatal) RhIg** |
| Rh alloimmunisation at delivery | 1357 (1 observational study) | ⨁◯◯◯ VERY LOW ^a,b^ | not estimable | 0 per 1,000 | 0 fewer per 1,000 (0 fewer to 0 fewer) |
| Rh alloimmunisation follow up: 6 months | 1357 (1 observational study) | ⨁◯◯◯ VERY LOW ^a,b^ | not estimable | 0 per 1,000 | 0 fewer per 1,000 (0 fewer to 0 fewer) |
| ***The risk in the intervention group** (and its 95% confidence interval) is based on the assumed risk in the comparison group and the **relative effect** of the intervention (and its 95% CI).   **CI:** Confidence interval | | | | | |
| **GRADE Working Group grades of evidence** **High certainty:** We are very confident that the true effect lies close to that of the estimate of the effect **Moderate certainty:** We are moderately confident in the effect estimate: The true effect is likely to be close to the estimate of the effect, but there is a possibility that it is substantially different **Low certainty:** Our confidence in the effect estimate is limited: The true effect may be substantially different from the estimate of the effect **Very low certainty:** We have very little confidence in the effect estimate: The true effect is likely to be substantially different from the estimate of effect | | | | | |

**Explanations**

a. No adjustment made for the detection and quantification of fetal-maternal hemorrhage, which was defined a priori as a variable that needed to be considered as part of an adjusted analysis.

b. No events in either group, so not evaluated.

## 2.5 Antenatal plus postnatal RhIg vs Postnatal RhIg

### 2.5.1. Results table

| **Author Year, Country (study design)** | **Timepoint** | **Ante- & Postnatal RhIg** | **Postnatal RhIg** | **Notes** |
| --- | --- | --- | --- | --- |
| **Rh alloimmunisation six months postpartum** | | | | |
| Tovey 1983 [13], UK (comparative cohort) | 6 months postpartum | 4/1238  (28 & 34 wks: 100 µg) | 19/1881  (100 µg) | Two women were thoughts to have 'naturally occurring' anti-D antibody in the ante- + postnatal group. |
| **Rh alloimmunisation postpartum or subsequent pregnancy** | | | | |
| Tiblad 2013 [14], Sweden (comparative cohort) | During the study period for antenatal group and in the 1^st^ trimester of a subsequent pregnancy for postnatal group | 24/9380  (28 wks: 250-300 µg) | 86/18546  (250-300 µg) | This includes all pregnancies regardless of baby Rh status, as we do not know the number of women in the postnatal only group who had Rh+ babies. 5104 pregnancies with Rh-positive babies in the ante- & postnatal group. Authors were contacted as they stated that they did not have the information for the reference cohort for Rh+ babies, however, they stated “In our population, about 60% of pregnant RhD negative women will have an RhD positive fetus/baby.” |
| Trolle 1989 [15], Denmark (comparative cohort) | 10 months after delivery or during their next pregnancy | 0/291  (28 wks: 300 µg) | 6/322 |  |
| **Sensitisation in the second pregnancy** | | | | |
| MacKenzie 1999 [7], UK (comparative cohort) | Second pregnancy | 12/3320  (28 & 34 wks: 500 IU) | 26/3146 | Also given at other at risk occasions during the antenatal period. Postnatal dose in both groups was 500 IU (i.e., 100 µg). |
| Thornton 1989 [16], UK (comparative cohort) | Second pregnancy with Rh+ baby | 1/604  (28 & 34 wks: 100 µg) | 9/582  (100 µg) | Same population as Tovey 1983, with follow-up data in 2^nd^ pregnancy. |

### 2.5.2. GRADE

#### Evidence profile

| **Certainty assessment** | | | | | | | **№ of patients** | | **Effect** | | **Certainty** |
| --- | --- | --- | --- | --- | --- | --- | --- | --- | --- | --- | --- |
| **№ of studies**  **[ref]** | **Study design** | **Risk of bias** | **Inconsistency** | **Indirectness** | **Imprecision** | **Other considerations** | **Antenatal and postnatal RhIg** | **Postnatal RhIg** | **Relative (95% CI)** | **Absolute (95% CI)** |  |
| Rh alloimmunisation 6 months postpartum | | | | | | | | | | | |
| 1  [13] | observational studies | extremely serious ^a^ | not serious | not serious | not serious ^b^ | none | 4/1238 (0.3%) | 19/1881 (1.0%) | **OR 0.39** (0.17 to 0.90) | **6 fewer per 1,000** (from 1 to 8 fewer) | ⨁◯◯◯ VERY LOW |
| Rh alloimmunisation postpartum or second pregnancy | | | | | | | | | | | |
| 2  [14,15] | observational studies | extremely serious ^a^ | not serious | serious ^c^ | not serious ^b^ | none | 24/9671 (0.2%) | 92/18868 (0.5%) | **OR 0.54** (0.37 to 0.80) | **2 fewer per 1,000** (from 1 to 3 fewer) | ⨁◯◯◯ VERY LOW |
| Sensitisation in second pregnancy | | | | | | | | | | | |
| 2  [7,16] | observational studies | extremely serious ^a^ | not serious | not serious | not serious ^b^ | none | 13/3924 (0.3%) | 35/3728 (0.9%) | **OR 0.38** (0.21 to 0.67) | **6 fewer per 1,000** (from 3 to 7 fewer) | ⨁◯◯◯ VERY LOW |

**CI:** Confidence interval; **OR:** Odds ratio

**Explanations**

a. No adjustment made for the detection and quantification of fetal-maternal hemorrhage, which was defined a priori as a variable that needed to be considered as part of an adjusted analysis.

b. Fewer events than the rule of thumb of 400 events, however, there is a sufficient number of participants (>2000). The relative CI does not cross the threshold of appreciable harm, and the absolute CI is narrow.

c. Tiblad 2013, contributing a weight of 94.3% (taken from Forest Plot Appendix 9.2: Figure 1) or representing 97.9% of the total population, includes women who had Rh negative and Rh positive babies. Among the 9380 in the ante- & postnatal group 5104 had Rh+ babies. We followed up with the authors to see if they had this information for the reference cohort, and although they did not, they stated that in their population, about 60% of pregnancy Rh-negative women will have a Rh-positive fetus/baby.

#### Summary of findings table

| **Outcomes** | **№ of participants (studies) Follow-up** | **Certainty of the evidence (GRADE)** | **Relative effect (95% CI)** | **Anticipated absolute effects** | |
| --- | --- | --- | --- | --- | --- |
|  |  |  |  | **Risk with postnatal RhIg** | **Risk difference with antenatal and postnatal RhIg** |
| Rh alloimmunisation 6 months postpartum | 3119 (1 observational study) | ⨁◯◯◯ VERY LOW ^a,b^ | **OR 0.39** (0.17 to 0.90) | 10 per 1,000 | 6 fewer per 1,000 (1 to 8 fewer) |
| Rh alloimmunisation postpartum or second pregnancy | 28539 (2 observational studies) | ⨁◯◯◯ VERY LOW ^a,b,c^ | **OR 0.54** (0.37 to 0.80) | 5 per 1,000 | 2 fewer per 1,000 (1 to 3 fewer) |
| Sensitisation in second pregnancy | 7652 (2 observational studies) | ⨁◯◯◯ VERY LOW ^a,b^ | **OR 0.38** (0.21 to 0.67) | 9 per 1,000 | 6 fewer per 1,000 (3 to 7 fewer) |
| ***The risk in the intervention group** (and its 95% confidence interval) is based on the assumed risk in the comparison group and the **relative effect** of the intervention (and its 95% CI).   **CI:** Confidence interval; **OR:** Odds ratio | | | | | |
| **GRADE Working Group grades of evidence** **High certainty:** We are very confident that the true effect lies close to that of the estimate of the effect **Moderate certainty:** We are moderately confident in the effect estimate: The true effect is likely to be close to the estimate of the effect, but there is a possibility that it is substantially different **Low certainty:** Our confidence in the effect estimate is limited: The true effect may be substantially different from the estimate of the effect **Very low certainty:** We have very little confidence in the effect estimate: The true effect is likely to be substantially different from the estimate of effect | | | | | |

**Explanations**

a. No adjustment made for the detection and quantification of fetal-maternal hemorrhage, which was defined a priori as a variable that needed to be considered as part of an adjusted analysis.

b. Fewer events than the rule of thumb of 400 events, however, there is a sufficient number of participants (>2000). The relative CI does not cross the threshold of appreciable harm, and the absolute CI is narrow.

c. Tiblad 2013, contributing a weight of 94.3% (taken from Forest Plot Appendix 9.2: Figure 1) or representing 97.9% of the total population, includes women who had Rh negative and Rh positive babies. Among the 9380 in the ante- & postnatal group 5104 had Rh+ babies. We followed up with the authors to see if they had this information for the reference cohort, and although they did not, they stated that in their population, about 60% of pregnancy Rh-negative women will have a Rh-positive fetus/baby.

### 2.5.3. Forest plot for Rh alloimmunisation postpartum or in second pregnancy

*Comparison: Antenatal plus postnatal RhIg vs Postnatal RhIg*


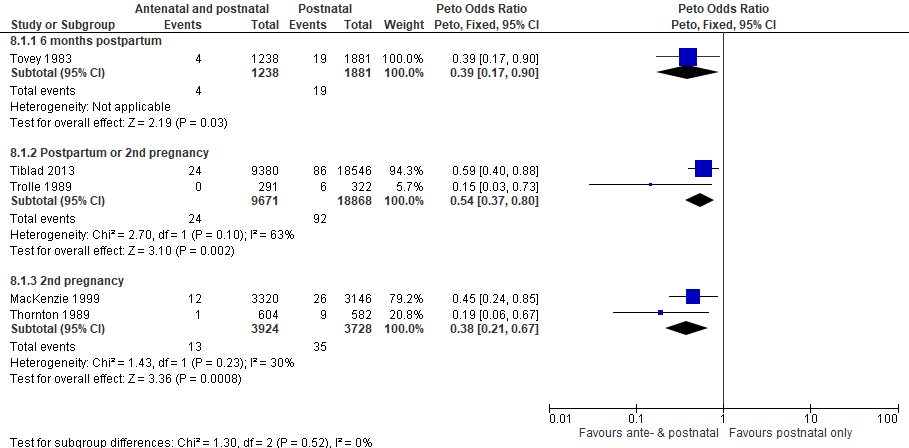


## 2.6 Shorter interval vs Longer interval between RhIg and delivery

### 2.6.1. Results table

| **Author Year** | **Timepoint** | **Interval between single-injection and delivery ≤8 weeks in most recent pregnancy** | **Interval between single-injection and delivery >8 to <16 weeks in most recent pregnancy** | **Notes** |
| --- | --- | --- | --- | --- |
| **Rh alloimmunisation six months postpartum** | | | | |
| Bowman & Pollack 1978 [17], Canada (comparative cohort) | 6 months postpartum | 0/163 | 2/923 | Rh negative primigravidas and multigravidas who had received antenatal and postnatal RhIg in all previous Rh-positive pregnancies and after all previous abortions. |
|  |  | 0/110 | 6/609 | Rh negative multigravidas who had received RhIg only after delivery or not at all after previous Rh-positive pregnancies or abortions. |

### 2.6.2. GRADE

#### Evidence profile

| **Certainty assessment** | | | | | | | **№ of patients** | | **Effect** | | **Certainty** |
| --- | --- | --- | --- | --- | --- | --- | --- | --- | --- | --- | --- |
| **№ of studies**  **[ref]** | **Study design** | **Risk of bias** | **Inconsistency** | **Indirectness** | **Imprecision** | **Other considerations** | **antenatal RhIg ≤8 weeks before delivery** | **antenatal RhIg >8 and <16 weeks before delivery** | **Relative (95% CI)** | **Absolute (95% CI)** |  |
| Rh alloimmunisation (Rh negative primigravidas and multigravidas who had received antenatal and postnatal RhIg in all previous Rh-positive pregnancies and after all previous abortions) (follow up: 6 months) | | | | | | | | | | | |
| 1  [17] | observational studies | extremely serious ^a^ | not serious | not serious | serious ^b^ | none | 0/163 (0.0%) | 2/923 (0.2%) | **OR 0.31** (0.01 to 14.95) | **1 fewer per 1,000** (from 2 fewer to 29 more) | ⨁◯◯◯ VERY LOW |
| Rh alloimmunisation (Rh negative multigravidas who had received RhIg only after delivery or not at all after previous Rh-positive pregnancies or abortions) (follow up: 6 months) | | | | | | | | | | | |
| 1  [17] | observational studies | extremely serious ^a^ | not serious | not serious | serious ^b^ | none | 0/110 (0.0%) | 6/609 (1.0%) | **OR 0.30** (0.03 to 2.83) | **7 fewer per 1,000** (from 10 fewer to 18 more) | ⨁◯◯◯ VERY LOW |

**CI:** Confidence interval; **OR:** Odds ratio

**Explanations**

a. No adjustment made for the detection and quantification of fetal-maternal hemorrhage, which was defined a priori as a variable that needed to be considered as part of an adjusted analysis.

b. Few events (less than rule of thumb of n=400 events) and small sample size (<2000 participants)

#### Summary of findings table

| **Outcomes** | **№ of participants (studies) Follow-up** | **Certainty of the evidence (GRADE)** | **Relative effect (95% CI)** | **Anticipated absolute effects** | |
| --- | --- | --- | --- | --- | --- |
|  |  |  |  | **Risk with antenatal RhIg >8 and <16 weeks before delivery** | **Risk difference with antenatal RhIg ≤8 weeks before delivery** |
| Rh alloimmunisation (Rh negative primigravidas and multigravidas who had received antenatal and postnatal RhIg in all previous Rh-positive pregnancies and after all previous abortions) follow up: 6 months | 1086 (1 observational study) | ⨁◯◯◯ VERY LOW ^a,b^ | **OR 0.31** (0.01 to 14.95) | 2 per 1,000 | 1 fewer per 1,000 (2 fewer to 29 more) |
| Rh alloimmunisation (Rh negative multigravidas who had received RhIg only after delivery or not at all after previous Rh-positive pregnancies or abortions) follow up: 6 months | 719 (1 observational study) | ⨁◯◯◯ VERY LOW ^a,b^ | **OR 0.30** (0.03 to 2.83) | 10 per 1,000 | 7 fewer per 1,000 (10 fewer to 18 more) |
| ***The risk in the intervention group** (and its 95% confidence interval) is based on the assumed risk in the comparison group and the **relative effect** of the intervention (and its 95% CI).   **CI:** Confidence interval; **OR:** Odds ratio | | | | | |
| **GRADE Working Group grades of evidence** **High certainty:** We are very confident that the true effect lies close to that of the estimate of the effect **Moderate certainty:** We are moderately confident in the effect estimate: The true effect is likely to be close to the estimate of the effect, but there is a possibility that it is substantially different **Low certainty:** Our confidence in the effect estimate is limited: The true effect may be substantially different from the estimate of the effect **Very low certainty:** We have very little confidence in the effect estimate: The true effect is likely to be substantially different from the estimate of effect | | | | | |

**Explanations**

a. No adjustment made for the detection and quantification of fetal-maternal hemorrhage, which was defined a priori as a variable that needed to be considered as part of an adjusted analysis.

b. Few events (less than the rule of thumb of n=400 events) and small sample size (<2000 participants)

### 2.6.3. Forest plot for Rh alloimmunisation six months postpartum

*Comparison: Shorter interval (≤ 8 week) vs Longer interval (>8 and <16 weeks) between RhIg and delivery*


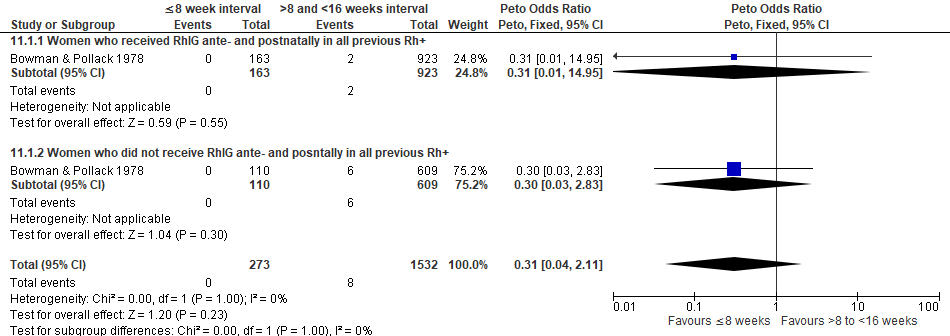


# 3. Abortion

## 3.1 RhIg vs No RhIg (placebo) given after spontaneous abortion

### 3.1.1. Results table

| **Author Year, Country (study design)** | **Timepoint** | **RhoGAM**  **(300 µg)** | **Placebo ^A^** | **Notes** |
| --- | --- | --- | --- | --- |
| **Rh alloimmunisation** | | | | |
| Visscher 1972 [18], USA (RCT) | 6 months post procedure | 0/19 | 0/29 | Given within 72 hours after a spontaneous complete abortion or operative termination of a spontaneous incomplete abortion. |

A Homologous gamma globulin (no demonstrable anti-Rho (D) antibody) 1 mL given by intramuscular injection

### 3.1.2. GRADE

#### Evidence profile

| **RhIg compared to No RhIg (placebo) given after spontaneous abortion for Rh alloimmunisation** | | | | | | | | | | | | |
| --- | --- | --- | --- | --- | --- | --- | --- | --- | --- | --- | --- | --- |
| **Certainty assessment** | | | | | | | **№ of patients** | | **Effect** | | **Certainty** |  |
| **№ of studies**  **[ref]** | **Study design** | **Risk of bias** | **Inconsistency** | **Indirectness** | **Imprecision** | **Other considerations** | **RhIg** | **No RhIg (placebo)** | **Relative (95% CI)** | **Absolute (95% CI)** |  |  |
| Rh alloimmunisation in women who experienced a spontaneous abortion (follow up: 6 months postpartum) | | | | | | | | | | | |  |
| 1  [18] | randomised trials | serious ^a^ | not serious | not serious | serious ^b^ | none | 0/19 (0.0%) | 0/29 (0.0%) | not estimable | - | ⨁⨁◯◯ LOW |  |

**CI:** Confidence interval; **OR:** Odds ratio

**Explanations**

a. Insufficient information to judge blinding of outcome assessment.

b. Few events (<rule of thumb of n=400 events) and small sample size (<2000).

#### Summary of Findings table

| **Outcomes** | **№ of participants (studies) Follow-up** | **Certainty of the evidence (GRADE)** | **Relative effect (95% CI)** | **Anticipated absolute effects** | |
| --- | --- | --- | --- | --- | --- |
|  |  |  |  | **Risk with No RhIg (placebo) given after spontaneous abortion** | **Risk difference with RhIg** |
| Rh alloimmunisation in women who experienced a spontaneous abortion (follow up: 6 months postpartum) | 48 (1 RCT) | ⨁⨁◯◯ LOW ^a,b^ | not estimable | 0 per 1,000 | **0 fewer per 1,000** (0 fewer to 0 fewer) |
| ***The risk in the intervention group** (and its 95% confidence interval) is based on the assumed risk in the comparison group and the **relative effect** of the intervention (and its 95% CI).   **CI:** Confidence interval; **OR:** Odds ratio | | | | | |
| **GRADE Working Group grades of evidence** **High certainty:** We are very confident that the true effect lies close to that of the estimate of the effect **Moderate certainty:** We are moderately confident in the effect estimate: The true effect is likely to be close to the estimate of the effect, but there is a possibility that it is substantially different **Low certainty:** Our confidence in the effect estimate is limited: The true effect may be substantially different from the estimate of the effect **Very low certainty:** We have very little confidence in the effect estimate: The true effect is likely to be substantially different from the estimate of effect | | | | | |

**Explanations**

a. Insufficient information to judge blinding of outcome assessment.

b. Few events (<rule of thumb of n=400 events) and small sample size (<2000).

##

## 3.2 Higher-dose vs Reduced-dose RhIg following first trimester vacuum abortion

### 3.2.1. Results table

| **Author Year, Country (study design)** | **Timepoint** | **Standard-dose (300 µg)** | **Reduced-dose (50 µg)** | **Notes** |
| --- | --- | --- | --- | --- |
| **Rh alloimmunisation** | | | | |
| Stewart 1978 [19], USA, (RCT) | 4-6 months follow-up | 0/64 | 0/691 |  |
| **Adverse events** | | | | |
| Stewart 1978 [19], USA (RCT) | After the procedure | 0/64 | 1/691 | Patient experienced nausea, dizziness, hypotension, bradycardia, which the physician felt was probably attributable to the abortion procedure, rather than the drug. |

### 3.2.2. GRADE

#### Evidence profile

| **Certainty assessment** | | | | | | | **№ of patients** | | **Effect** | | **Certainty** |
| --- | --- | --- | --- | --- | --- | --- | --- | --- | --- | --- | --- |
| **№ of studies**  **[ref]** | **Study design** | **Risk of bias** | **Inconsistency** | **Indirectness** | **Imprecision** | **Other considerations** | **Standard dose** | **Reduced dose** | **Relative (95% CI)** | **Absolute (95% CI)** |  |
| **Rh alloimmunisation (follow up: 4 to 6 months)** | | | | | | | | | | | |
| 1  [19] | randomised trials | very serious ^a^ | not serious | not serious | serious ^b^ | none | 0/64 (0.0%) | 0/691 (0.0%) | not estimable | - | ⨁◯◯◯ VERY LOW |

**CI:** Confidence interval

**Explanations**

a. 26.5% participants lost to follow-up, with significant differences between those who completed follow-up and lost to follow-up in higher gravidity, parity, and frequency of previous abortions.

b. Few events (<rule of thumb of n=400 events) and small sample size (<2000 participants).

#### Summary of Finding table

| **Outcomes** | **№ of participants (studies) Follow-up** | **Certainty of the evidence (GRADE)** | **Relative effect (95% CI)** | **Anticipated absolute effects** | |
| --- | --- | --- | --- | --- | --- |
|  |  |  |  | **Risk with Reduced-dose RhIg** | **Risk difference with Standard-dose RhIg** |
| Rh alloimmunisation follow up: 4 to 6 months | 755 (1 RCT) | ⨁◯◯◯ VERY LOW ^a,b^ | not estimable | 0 per 1,000 | **0 fewer per 1,000** (0 fewer to 0 fewer) |
| ***The risk in the intervention group** (and its 95% confidence interval) is based on the assumed risk in the comparison group and the **relative effect** of the intervention (and its 95% CI).   **CI:** Confidence interval | | | | | |
| **GRADE Working Group grades of evidence** **High certainty:** We are very confident that the true effect lies close to that of the estimate of the effect **Moderate certainty:** We are moderately confident in the effect estimate: The true effect is likely to be close to the estimate of the effect, but there is a possibility that it is substantially different **Low certainty:** Our confidence in the effect estimate is limited: The true effect may be substantially different from the estimate of the effect **Very low certainty:** We have very little confidence in the effect estimate: The true effect is likely to be substantially different from the estimate of effect | | | | | |

**Explanations**

a. 26.5% participants lost to follow-up, with significant differences between those who completed follow-up and lost to follow-up in higher gravidity, parity, and frequency of previous abortions.

b. Few events (<rule of thumb of n=400 events) and small sample size (<2000 participants).

# 4. Amniocentesis

## 4.1 RhIg vs No RhIg after amniocentesis

### 4.1.1. Results table

| **Author Year, Country (study design)** | **Timepoint** | **RhIg** | **No RhIg** | **Notes** |
| --- | --- | --- | --- | --- |
| **Rh alloimmunisation most recent pregnancy** | | | | |
| MRC report 1978 [20], UK (comparative cohort) | From most recent pregnancy | 0/59 | 3/58 | No information provided on dose of anti-D. |

### 4.1.2. GRADE

#### Evidence profile

| **Certainty assessment** | | | | | | | **№ of patients** | | **Effect** | | **Certainty** |
| --- | --- | --- | --- | --- | --- | --- | --- | --- | --- | --- | --- |
| **№ of studies**  **[ref]** | **Study design** | **Risk of bias** | **Inconsistency** | **Indirectness** | **Imprecision** | **Other considerations** | **RhIg** | **No RhIg** | **Relative (95% CI)** | **Absolute (95% CI)** |  |
| Rh alloimmunisation (among women who received amniocentesis) | | | | | | | | | | | |
| 1  [20] | observational studies | extremely serious ^a^ | not serious | not serious | serious ^b^ | none | 0/59 (0.0%) | 3/58 (5.2%) | **OR 0.13** (0.01 to 1.26) | **45 fewer per 1,000** (from 51 fewer to 13 more) | ⨁◯◯◯ VERY LOW |

**Explanations**

a. No adjustment made for the detection and quantification of fetal-maternal hemorrhage, which was defined a priori as a variable that needed to be considered as part of an adjusted analysis.

b. Few events (<rule of thumb of n=400 events) and small sample size (<2000 participants).

#### Summary of findings table

| **Outcomes** | **№ of participants (studies) Follow-up** | **Certainty of the evidence (GRADE)** | **Relative effect (95% CI)** | **Anticipated absolute effects** | |
| --- | --- | --- | --- | --- | --- |
|  |  |  |  | **Risk with no RhIg** | **Risk difference with RhIg** |
| Rh alloimmunisation after amniocentesis | 117 (1 observational study) | ⨁◯◯◯ VERY LOW ^a,b^ | **OR 0.13** (0.01 to 1.26) | 52 per 1,000 | 45 fewer per 1,000 (51 fewer to 13 more) |

**Explanations**

a. No adjustment made for the detection and quantification of fetal-maternal hemorrhage, which was defined a priori as a variable that needed to be considered as part of an adjusted analysis.

b. Few events (<rule of thumb of n=400 events) and small sample size (<2000 participants).

### 4.1.3 Forest plot for Rh immunisation from most recent pregnancy

*Comparison: RhIg vs No RhIg after amniocentesis*


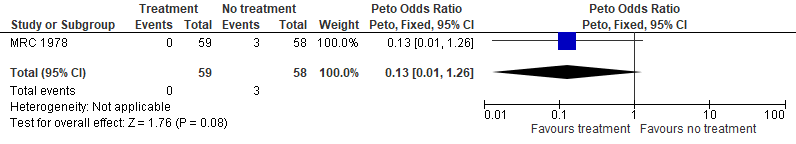


# 5. Intramuscular vs Intravenous

## 5.1. Antenatal RhIg given intramuscularly vs Antenatal RhIg given intravenously

### 5.1.1. Results table

| **Author Year, Country (study design)** | **Timepoint** | **Rhophylac 300 µg given intramuscularly** | **Rhophylac 300 µg given intravenously** | **Notes** |  |
| --- | --- | --- | --- | --- | --- |
| **Rh alloimmunisation** | | | | | |
| Bichler 2003 [21], ^A,B^ Germany (RCT) | 6 months | 0/8 | 0/6 |  |  |
| MacKenzie 2004 [22],^B^ UK (RCT) | 9 months | 1/216 | 0/216 | The one woman was no longer positive at 11.5 months. |  |
| **Adverse events** | | | | | |
| Bichler 2003 [21],^A,B^ Germany (RCT) | Not reported | 3/8  (Three women experienced four adverse events) | 2/6  (Two women experienced three adverse events) | As it was not clear how adverse events were collected and reporting was poor, the level of certainty was not evaluated. |  |
| MacKenzie 2004 [22],^B,C^ UK (RCT) | After antenatal dose | One woman experienced mild pain at the injection site, which persisted for 1 week (withdrew from study). One woman experienced mild itching around the injection site for 1 hour. | One woman experienced a headache for a few hours on the day of antenatal dose (did not happen after postnatal injection). One woman experienced mild soreness for 4 days at the site of injection. | As it was not clear how adverse events were collected and reporting was poor, the level of certainty was not evaluated. |  |

A The administration of the postnatal prophylaxis was not randomised, but was up to the discretion of the investigator.

B Given at 28 weeks gestation and within 72 hours after delivery of an RhD-positive child

C Once randomised to a treatment group, any further study medication required during the study was given within the same treatment group.

### 5.1.2. GRADE

#### Evidence profile

| **Intramuscular compared to Intravenous given during pregnancy for Rh alloimmunisation** | | | | | | | | | | | | |
| --- | --- | --- | --- | --- | --- | --- | --- | --- | --- | --- | --- | --- |
| **Certainty assessment** | | | | | | | **№ of patients** | | **Effect** | | **Certainty** |  |
| **№ of studies**  **[ref]** | **Study design** | **Risk of bias** | **Inconsistency** | **Indirectness** | **Imprecision** | **Other considerations** | **Intramuscular** | **Intravenous** | **Relative (95% CI)** | **Absolute (95% CI)** |  |  |
| Rh alloimmunisation at ≥6 months postpartum | | | | | | | | | | | |  |
| 2  [21,22] | randomised trials | very serious ^a^ | not serious | not serious | serious ^b^ | none | 1/224 (0.4%) | 0/222 (0.0%) | **OR 7.39** (0.15 to 372.38) | **0 fewer per 1,000** (from 0 fewer to 0 fewer) | ⨁◯◯◯ VERY LOW |  |

**CI:** Confidence interval; **OR:** Odds ratio

**Explanations**

a. Method of randomisation unclear for one study, allocation concealment not done or unclear, and insufficient information to provide a judgement for blinding of outcome assessment.

b. Few events (<rule of thumb of n=400 events) and small sample size (<2000 participants).

#### Summary of Findings table

| **Outcomes** | **№ of participants (studies) Follow-up** | **Certainty of the evidence (GRADE)** | **Relative effect (95% CI)** | **Anticipated absolute effects** | |
| --- | --- | --- | --- | --- | --- |
|  |  |  |  | **Risk with Intravenous given during pregnancy** | **Risk difference with Intramuscular** |
| Rh alloimmunisation at ≥6 months postpartum | 446 (2 RCTs) | ⨁◯◯◯ VERY LOW ^a,b^ | **OR 7.39** (0.15 to 372.38) | 0 per 1,000 | **0 fewer per 1,000** (0 fewer to 0 fewer) |
| ***The risk in the intervention group** (and its 95% confidence interval) is based on the assumed risk in the comparison group and the **relative effect** of the intervention (and its 95% CI).   **CI:** Confidence interval; **OR:** Odds ratio | | | | | |
| **GRADE Working Group grades of evidence** **High certainty:** We are very confident that the true effect lies close to that of the estimate of the effect **Moderate certainty:** We are moderately confident in the effect estimate: The true effect is likely to be close to the estimate of the effect, but there is a possibility that it is substantially different **Low certainty:** Our confidence in the effect estimate is limited: The true effect may be substantially different from the estimate of the effect **Very low certainty:** We have very little confidence in the effect estimate: The true effect is likely to be substantially different from the estimate of effect | | | | | |

**Explanations**

a. Method of randomisation unclear for one study, allocation concealment not done or unclear, and insufficient information to provide a judgement for blinding of outcome assessment.

b. Few events (<rule of thumb of n=400 events) and small sample size (<2000 participants).

### 5.1.3. Forest plot for Rh alloimmunisation at 6-9 months postpartum

*Comparison: Antenatal administration given intramuscularly vs Antenatal administration given intravenously*


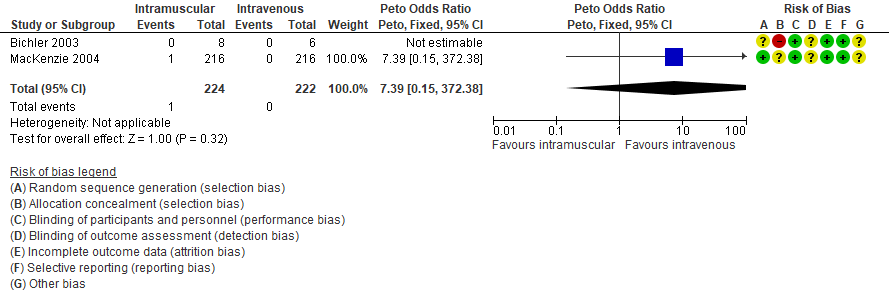


**Risk of bias legend**: (A) Random sequence generation (selection bias); (B) Allocation concealment (selection bias); (C) Blinding of participants and personnel (performance bias); (D) Blinding of outcome assessment (detection bias); (E) Incomplete outcome data (attrition bias); (F) Selective reporting (reporting bias); (G) Other bias
