# Supplementary material for: Antenatal and postpartum prevention of Rh alloimmunization: A systematic review and GRADE analysis
Source: PLoS One. 2020 Sep 10;15(9):e0238844. doi: 10.1371/journal.pone.0238844 (PMC7482964; doi:10.1371/journal.pone.0238844)
Supplement: Attachment — Submitted filename: Response to Reviewers.docx [file pone.0238844.s008.docx]

**Response to peer-review feedback**

Reviewer #1: Antenatal and Postpartum Prevention of Rh Alloimmunization: A Systematic Review and GRADE analysis

Thank you for the opportunity to review this systematic review and GRADE analysis. The body of work is extensive and comprehensive, addressing an issue of enduring importance in obstetrics. The subtleties of timing and dosage of “Anti-D” during pregnancy are well expounded. The review provides an excellent overview of the evidence to inform clinical practice guidelines, albeit identifying why there is variation in these, given the heterogeneity of studies.

**Response**: Thank you for your time in reviewing our manuscript and for providing feedback. We have made the changes as suggested below.

I have only extremely minor suggestions:

Line 40 “where” is “were”

**Response**: Thank you for catching this, it has been corrected.

Table 2: “Medical Research Council 1974,[41] RCT
Funding: not Reported”
Wouldn’t this be MRC-funded?

**Response**: Good point. I imagine this is likely the case, but it is not explicitly stated in the publication. I have kept not reported, but added (likely MRC) after.

Table 3: add the word “be” to Bowman & Pollock, participant demographics, “… in this group may [be] due …”

**Response**: Thank you, this has been corrected.

Table 3: MRC by Working Party on Amniocentesis… participant demographics, replace “subjects” with “women”.

**Response**: OK, this has been changed from subjects to women.
